# Autophagy characteristics and establishment of autophagy prognostic models in lung adenocarcinoma and lung squamous cell carcinoma

**DOI:** 10.1371/journal.pone.0266070

**Published:** 2022-03-25

**Authors:** Zhubei Chen, Hui Xiong, Hao Shen, Qingsheng You

**Affiliations:** 1 Department of Cardiothoracic Surgery, Affiliated Hospital of Nantong University, Nantong, China; 2 Nantong University Medical School, Nantong, China; University of Wisconsin, UNITED STATES

## Abstract

**Background:**

Non-small cell lung cancer (NSCLC), which makes up the majority of lung cancers, remains one of the deadliest malignancies in the world. It has a poor prognosis due to its late detection and lack of response to chemoradiaiton. Therefore, it is urgent to find a new prognostic marker.

**Methods:**

We evaluated biological function and immune cell infiltration in lung adenocarcinoma (LUAD) and lung squamous cell carcinoma (LUSC) patients from TCGA and GEO databases between different clusters based on autophagy related hub genes. Autophagy scores were used to assess the degree of autophagy in each individual by using component analysis.

**Results:**

Three different clusters were obtained. Gene set variation analysis, single-sample gene set enrichment analysis and survive analysis showed differences among these three clusters. We demonstrated that the autophagy score of each patient could predict tumor stage and prognosis. Patients with a high autophagy score had a better prognosis, higher immune infiltration, and were more sensitive to immunotherapy and conventional chemotherapy.

**Conclusion:**

It was uncovered that autophagy played an irreplaceable role in NSCLC. Quantified autophagy scores for each NSCLC patient would help guide effective treatment strategies.

## Introduction

Lung cancer is still one of the most difficult problems, and it is one of the leading causes of cancer death worldwide [1]. Histopathologically, lung cancer is subdivided into small cell lung cancer (SCLC) and non-small cell lung cancer (NSCLC). Among them, NSCLC accounts for 85% of all lung cancer cases, and it is mainly composed of lung squamous cell carcinoma (LUSC) and lung adenocarcinoma (LUAD) [2]. With the research and exploration of related technologies, NSCLC has made undeniable progress in surgical treatment, chemotherapy and immunotherapy. However, the 5-year survival rate of NSCLC is still poor in China. Mainly due to the lack of early diagnosis and classification treatment. Therefore, the only way to alleviate this situation is to find a new prognostic assessment method to guide individualized treatment of NSCLC patients [3].

Studies have shown that autophagy is associated with the occurrence and progression of lung cancer [4]. There are three types of autophagy: macroautophagy, microautophagy and chaperone-mediated autophagy. Macroautophagy is the main form of autophagy, which is what we usually refer to as the type of autophagy. This is a highly conserved catabolic process that degrades damaged or dysfunctional proteins or organelles through lysosomes [5,6]. The target substrate is first separated by the phagophore (a unique membrane structure) into a double-membrane autophagosome. Then, the autophagosome sends the cargo for degradation through fusing with endosomes and lysosomal vesicles to mature into amphisomes and autolysosomes. Finally, the degradation products will be recycled for cell synthesis biological reactions. Macroautophagy relies on the production of autophagosomes. On the contrary, during microautophagy, the lysosome use its own membrane to swallow substrates and degrade them. In chaperone-mediated autophagy, after the target protein with a specific sequence is recognized by the chaperone, it binds to the special receptor on the lysosomal membrane—the lysosomeassociated membrane glycoprotein type 2A (Lamp2A), and enters the lysosome to be degraded. However, there are pros and cons to autophagy. In the initial stage of cancer, it can swallow cancer cells and protect normal cells from tumor invasion. In the advanced stage of cancer, it can also provide energy and nutrition for cancer cells to promote their growth [7]. Related research found that high expression of autophagy-related gene 10 was associated with poor prognosis of lung cancer. SKI like proto-oncogene promotes tumorigenesis and immune escape of NSCLC by up-regulating the Tafazzin, Phospholipid-Lysophospholipid Transacylase/autophagy axis. Casein Kinase 1 Alpha 1 inhibits lung tumor growth by stabilizing PTEN and inducing autophagy [8–10]. These studies had proved that autophagy was involved in lung cancer, and suggested that autophagy-related genes might be considered as prognostic markers in lung cancer.

In this study, we explored the relationship between autophagy-related genes (ARGs) and the prognosis of LUAD and LUSC, and established an autophagy scoring system to evaluate patients. Based on the score, we could predict the prognosis of patients and guide patients to make the best treatment choice.

## Materials and methods

### Data acquisition and pre-processing

The gene expression profiles and associated clinical information of lung cancer were available from The Cancer Genome Atlas (TCGA) database (https://portal.gdc.cancer.gov/) on April 11th, 2021. The database includes clinical data of various human cancers (including tumor subtypes), which is an important data source for cancer researchers. The download gene expression profiles met the following conditions: (1) the primary site was “bronchus and lung”; (2) the program was “TCGA”; (3) the disease type were “adenomas and adenocarcinomas” and “squamous cell neoplasms”; (4) the data category was “transcriptome profiling”; (5) the data type was “Gene Expression Quantification”; (6) the workflow type was “HTSeq-FPKM”. The TCGA samples include 103 normal samples and 999 tumor samples, of which 502 lung squamous carcinoma samples and 497 lung adenocarcinoma samples are among the tumor samples. In addition, we downloaded series matrix files and platform files of three datasets (GSE41271, GSE42127, GSE37745) from the Gene Expression Omnibus (GEO) database (https://www.ncbi.nlm.nih.gov/geo/), a database for storing chips, next-generation sequencing, and other high-throughput sequencing data. Patients diagnosed with LUAD or LUSC were selected. The basic information of the three datasets is provided in Table 1 [11].

**Table 1:** Basic information of four datasets mentioned in this article.

Data set source	Platform	Pathology	Stage	Gender	Number of patients
TCGA: LUAD+LUSC	Illumina RNAseq	LUAD:497 LUSC:502	I/II:782 III/IV:196	Female:395 Male:595	999
GEO: GSE41271	GPL6884 Illumina HumanWG-6 v3.0 expression beadchip	LUAD:184 LUSC:81	I/II:178 III/IV:87	Female:121 Male:144	275
GEO: GSE42127	GPL6884 Illumina HumanWG-6 v3.0 expression beadchip	LUAD:132 LUSC:43	I/II:144 III/IV:31	Female:132 Male:43	176
GEO: GSE37745	GPL570 Affymetrix Human Genome U133 Plus 2.0 Array	LUAD:106 LUSC:66 LCC^a^:24	NA	Female:89 Male:107	196

Table 1 showed the basic information of four datasets. Due to lacking of information about some patients, the total number of patients in the previous classification was less than the total number of patients in the whole datasets.

^a^ LCC, large cell lung cancer.

We also download somatic mutation data of LUAD and LUSC from TCGA database. The data were acquired according to the following conditions: (1) the data category was “simple nucleotide variation”; (2) the data type was “Masked Somatic Mutation”; (3) the workflow type was “VaScan2 Variant Aggregation and Masking”. Meanwhile the copy number (gene-level) data of GDC TCGA-LUAD and TCGA-LUSC were got from UCSC Xena (http://xena.ucsc.edu/) for following Copy Number Variation (CNV) analysis [12].

Gene expression profiles from TCGA were converted to the matrix of standard human gene expression of each sample. We took the average of the expression of the same gene. Then the FPKM (Fragments Per Kilobase Million) data were converted into the TPM (Transcripts Per Million) data [13]. The two steps were using the R package “limma”. At the same time, we extracted clinical information files (including id, age, gender, survival status, overall survival time (OS), stage and pathology) and Gene expression file (including id and probe name) from the two series of matrix files downloaded from GEO database respectively. According to the platform file, we use Perl to batch convert the probe names in the gene expression files into standard human gene names. The batch effect caused by non-biotechnology deviations in these three datasets was rectified by using the “combat” function in the R package “SVA” [14]. If the pathology of the sample was normal or the sample lacking any clinical information such as sample id, age, gender, survival status, overall survival time (OS) and pathology would be deleted in the clinical information file. The merger cohort consisting of TCGA-LUAD, TCGA-LUSC, GSE41271, and GSE42127 was used as the training set, and GSE37745 was used as the validation set. R (version 4.0.4) and strawberry-perl (version 5.30.2.1) were used to process data.

### PPI network construction and acquirement of autophagy-related hub genes (ARHGs)

Autophagy-related genes (ARGs) were obtained from Human Autophagy Database (http://www.autophagy.lu/index.html). A PPI network of ARGs was constructed by using the STRING database (https://string-db.org/), where minimum required interaction score was set to 0.95 and free nodes were deleted. Then the network was visualized by using Cytoscape software (version 3.7.2), in which the CytoNCA plug-in (version2.1.6) was used to calculate scores of each node’s BC (Betweenness), CC (Closeness), DC (Degree), EC (Eigenvector), LAC (Local Average Connectivity-based method) and NC (Network). Only the nodes that were higher than the median value of each score at the same time would be retained, and node genes were called ARHGs [15].

### Unsupervised clustering for ARHGs

Intersection ARHGs were extracted from three integrated datasets. We applied unsupervised clustering analysis to classify the samples based on the expression of the intersection of ARHGs. We used the R package “ConsensusClusterPlus” to repeat the above steps 1000 times for guaranteeing the stability of classification. The number of clusters and stability was determined by the clustering algorithm [16].

### Gene set variation analysis (GSVA)

GSVA, an unsupervised gene set enrichment method which was used to estimate the differences in the activity of different pathways between high and low score groups. In order to reveal the differences in biological pathways between different clusters based on ARHGs, we downloaded the “c2.cp.kegg.v7.4.symbols” from Gene Set Enrichment Analysis (http://www.gsea-msigdb.org/gsea/index.jsp). The R package “GSVA” was used to calculate the score of separate pathways in each sample, where the parameter of “min.sz” was set to 10, “max.sz” was set to 500 and “parallel.sz” was set to 1. Subsequently, differential analysis of pathways was carried out by using the R package “limma”. The pathway adjusted P < 0.05 was regarded as having differentially expressed pathway [17,18].

### Immune infiltration analysis

We downloaded the gene set for marking each TME infiltrating immune cell type which was based on Charoentong’s research on immunity from “The Cancer Immunome Atlas” (http://icbi.at/TCIA/SupplementaryTables.pdf). Single-sample gene set enrichment analysis (ssGSEA) method was carried out to calculate the relative abundance of each immune cell infiltration in each sample in the TME (tumor microenvironment) of lung cancer by R package “GSVA” [19].

### Screening of differential expressed genes (DEGs) between ARHG-clusters

In order to discover DEGs related to autophagy, R package “limma” and “VennDiagram” were used to identify DEGs between ARHG-clusters, of which DEGs whose corrected p-value was less than 0.001 were retained [20].

### Function and pathways analysis of DEGs

GO (Gene Ontology) and KEGG (Kyoto Encyclopedia of Genes and Genomes) were conducted to analyse the function and pathways of DEGs by utilizing the R packages “org.Hs.eg.db”. The filter condition was that P-value and q-value were both less than 0.05. According to the number of gene enrichment, sorting from largest to smallest, we took the top 30 KEGG pathways and the top 30 GOs terms including the top 10 BP (biological process) terms, 10 CC (cellular component) terms and 10 MF (molecular function) terms [21].

### Establishment of ATscore(autophagy score) System

In order to evaluate the degree of autophagy in each lung cancer patient, we constructed a set of autophagy scoring system through autophagy genes related to prognosis which we called ATscore. This system was set up through the following steps:

According to the patient’s survival status and survival time, univariate Cox analysis of DEGs was performed to find genes related to prognosis and only p-values<0.05 were regarded as significant [22]. Patients were structured in several groups for further analysis by using an unsupervised clustering method for analyzing DEGs related to prognosis. The parameters were similar to ARHGs. The cluster was named geneCluster. At the same time, we analyzed the differences expression of ARHGs between geneClusters.

Then we used the PCA (principal component analysis) method to construct a scoring system. Components 1 and 2 would be chosen to form this scoring system [23]. We used a method similar to GGI [24] to define the ATscore of each patient:

$$ATscore=\sum {\left(PC1+PC2\right)}_{i}$$

Where i represented the expression of DEGs related to prognosis.

### Correlation between ATscore and clinical traits

In order to know the relationship between ATscore and clinical characters, we analyzed ATscore with each patient’s gender, age, stage, pathology, and survival status, and verified the relationship between ATscore and survival among different independent clinical traits. Meanwhile we analyzed patients with the same clinical traits separately to eliminate the interference of clinical traits on the results. Univariate Cox analysis and multivariate cox analysis were used to explore the relationship between ATscore and survival.

### Treatment prediction of LUAD and LUSC based on ATscore

To know the immunogenicity of patients with high and low ATscore in LUAD and LUSC. We obtained the immunophenotypic score (IPS) of TCGA-LUAD and TCGA-LUSC patients from The Cancer Immunome Database (TCIA, https://tcia.at/home). Then the differences of IPS between high ATscore group and low ATscore group in LUAD and LUSC were analyzed [25].

We use R package “pRRophetic” to predict the half-maximal inhibitory concentration (IC50) of five common chemotherapy drugs (cisplatin, gemcitabine, paclitaxel, vinorelbine, and methotrexate) used to treat LUAD and LUSC [26]. The prediction of sensitivity of LUAD and LUSC patients to these five chemotherapy drugs was based on Genomics of Drug Sensitivity in Cancer (GDSC, https://www.cancerrxgene.org/).

To further verify the accuracy of our model, we calculated the ATscore of each patient in GSE37745 according to the previous methods (unsupervised cluster analysis based on ARHGs, acquisition of prognostic-related DEGs, PCA analysis), and analyzed the survival of patients between the high and low ATscore groups.

### Statistical analysis

The wilcox test was used to compare the differences between the two groups, and the Kruskal-Wallis test was used to compare the differences between three groups and above. By utilizing the "Surv-Cutpoint" function in the R package “Survminer”, the patients were divided into high ATscore group and low ATscore group. Tumor mutation burden (TMB) is the total number of gene coding errors, base substitutions, gene insertion or deletion errors per million bases in tumor cells. The TMB was also in the same method [27]. The survival time of the patient was evaluated by KM (Kaplan-Meier) survival analysis, and the different groups were compared by using a log-rank test. We used the "Pearson" method to calculate correlations coefficients between ARHGs, and P-value less than 0.0001 was thought meaningful. The hazard ratios (HR) for ARHGs and DEGs related to prognosis were calculated by univariate cox analysis. R package “Corrplot” was performed to visualize the relationships which were calculated by the “corr.mtest” function between Autophagy-score and immune cells. The relationship coefficient between TMB and Autophagy-score was obtained by the “Spearman” method. We used R package “RCircos” [28] to display the copy number variation of ARHGs in TCGA-LUAD and TCGA-LUSC on 23 human chromosomes and used “Waterfull” function in R package “maftools” to show the mutation status in TCGA-LUAD and TCGA-LUSC, as well as the mutation status between high and low ATscore groups. The calculation of all data was done through R (version 4.0.4) [29]. The R packages used in this study and their usage methods could be obtained from "bioconduction" (http://bioconductor.org/packages/release/BiocViews.html#___Software).

## Results

### Mutations of ARGs in LUAD and LUSC

**Fig 1:**
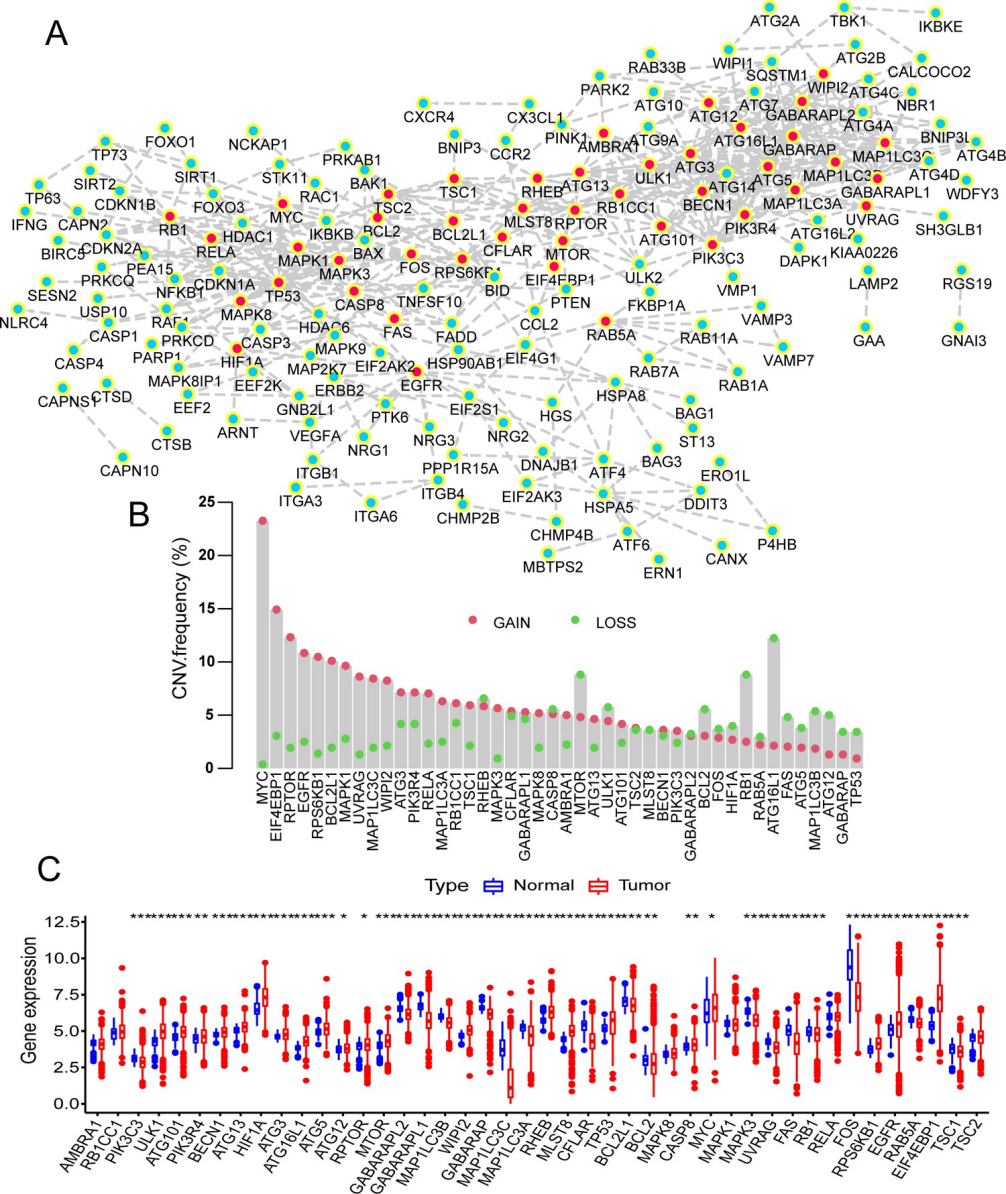
The generation of ARHGs and the copy number and expression in TCGA database. (A) Construction of the PPI network of ARGs with interaction score more than 0.95. ARHGs were marked in red, and non-ARHGs were marked in green. (B) The CNV mutation frequency of ARHGs in LUAD and LUSC in TCGA database. The height of the column represented the frequency of mutation. Deletions were indicated by green dots, and amplifications were indicated by red dots. (C) The expression of ARHGs between lung cancer tissues and normal tissues in LUAD and LUSC in TCGA database. Normal tissues were shown in blue while tumor tissues were shown in red. The upper and lower ends of the box indicated the interquartile range of expression value of ARHGs, and the horizontal line in the box indicated the median value of expression. The points outside the box represented outliers. The asterisk indicated the P-value (*P<0.05, **P<0.01, ***P<0.001).

**Fig 2:**
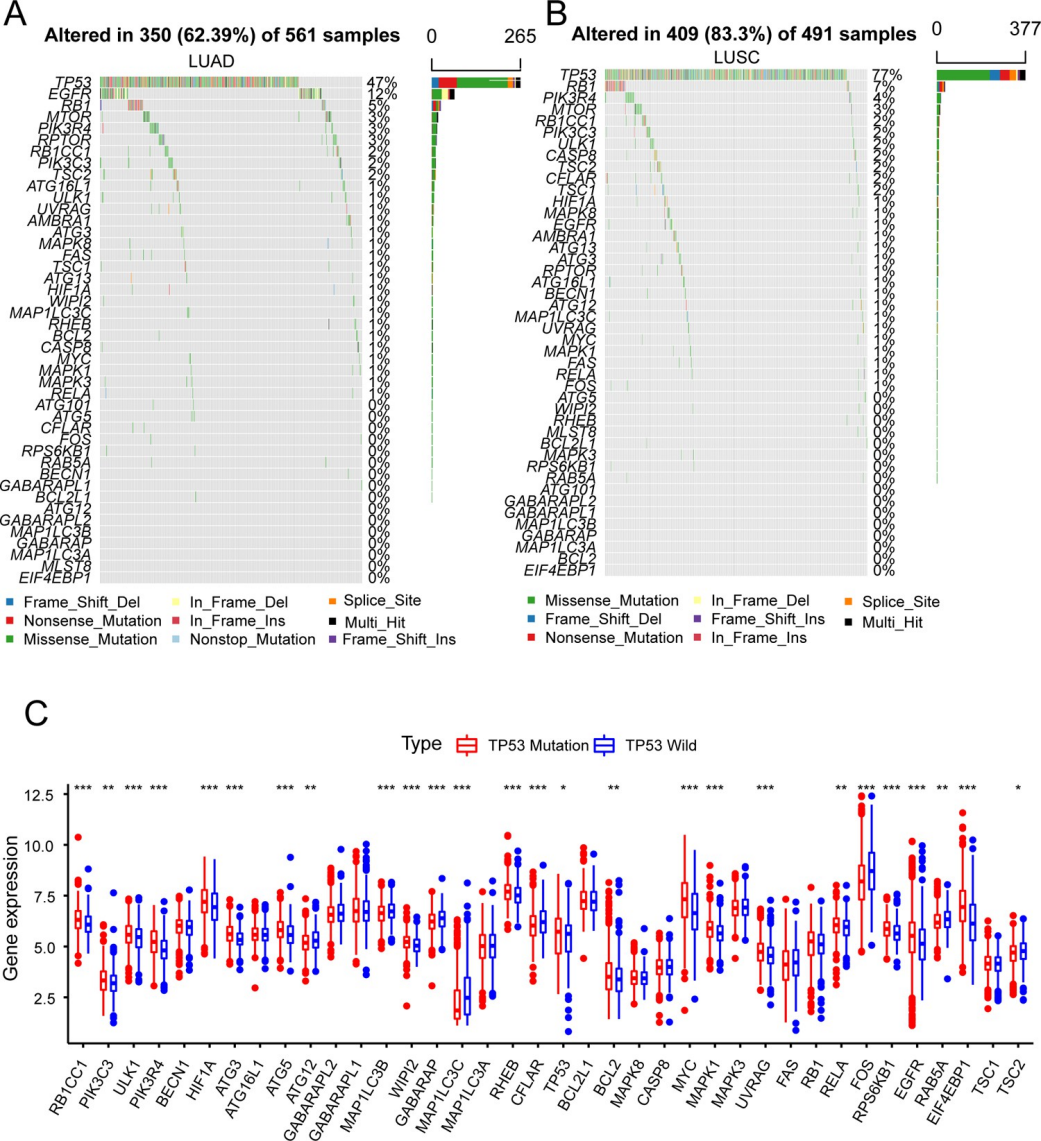
The mutation of ARHGs in TCGA database. (A~B) The mutation frequency of ARHGs in patients with lung cancer in LUAD and LUSC in TCGA database. Each column represented each patient. The number on the right represented the mutation frequency of each ARHG, and the bar graph indicated the proportion of each base mutation. (C) The expression of ARHGs between TP53 mutation group and TP53 wild group in LUAD and LUSC in TCGA database. The TP53 mutation group was marked in red, and the TP53 wild group was marked in blue. The upper and lower ends of the box indicated the interquartile range of ARHGs expression, and the horizontal line in the box indicated the median value of ARHGs expression. The points outside the box indicated outliers. The asterisk showed the P-value (*P<0.05, **P<0.01, ***P<0.001).

In order to know the central role of ARGs, we screened 44 ARHGs from 232 ARGs, and the specifics would be shown in Fig 1A. First, we assessed the frequency of total CNV of ARHGs in TCGA data set. It was found that the CNV changes of ARHGs were universal, most of the genes were in the increase of copy number, of which the MYC gene copy number increase was the most active, while a few genes were in the state of copy number loss, and the ATG16L1 gene copy number loss was the most (Fig 1B). Secondly, we performed somatic mutation analysis on TCGA-LUAD and TCGA-LUSC respectively. 350 samples out of 561 LUAD samples were mutated, with a mutation rate of 62.39%. Among them, TP53 had the highest mutation frequency, followed by EGFR, while ATG101 and other genes had no mutations (Fig 2A). In LUSC, 409 out of 491 samples were mutated, and the mutation frequency was 83.3%. Interestingly, the highest mutation rate was also TP53. The second highest was RB1 and no mutations in ATG5 etc. (Fig 2B). Correlation analysis show that most ARGHs were positively correlated, and a few were negatively correlated such as EIF4EBP1 and MAP1LC3C, EIF4EBP1 and CFLAR (S1A Fig). Further analysis of the genes with the highest mutation frequency showed that the expression levels of most genes in the TP53 mutant group were higher than those in the wild group, such as TP53, EGFR, and MYC. Some genes were expressed more highly in the wild group, such as MAP1LC3B, CFLAR and FOS (Fig 2C) [30]. In order to determine whether the above-mentioned mutations affected the expression of ARHGs in patients who were LUSC or LUAD, we compared the expression levels of ARHGs in normal people and patients with LUAD or LUSC in the TCGA database. Compared with normal tissues, most ARHGs are highly expressed in lung cancer tissues. Interestingly, we found that ARHGs with active copy number increase (e.g. MYC, EIF4EBP1, EGFR) and ARHGs with active copy number loss (e.g. ATG16L1, MTOR, ULK1) also showed high expression in lung cancer tissues (Fig 1C). Therefore, we speculated that the change of CNV was an element that led to the disorder of ARHGs expression in tumor tissues. Through the above analysis, ARHGs are highly heterogeneous in normal tissues and lung cancer tissues. Through the above analysis, ARHGs had a high degree of heterogeneity in normal tissues and lung cancer tissues, which indicated that the expression imbalance of ARGs played a very important role in the development of lung cancer [31].

### The unsupervised clustering based on ARHGs

LUAD and LUSC from the TCGA database and two cohorts (GSE41271, GSE42127) from the GEO database were formed into a new cohort (1419 lung cancer patients) which we named merger cohort. We intersected the genes in the merger cohort with 44 ARHGs, and finally got 38 ARHGs [32].

**Fig 3:**
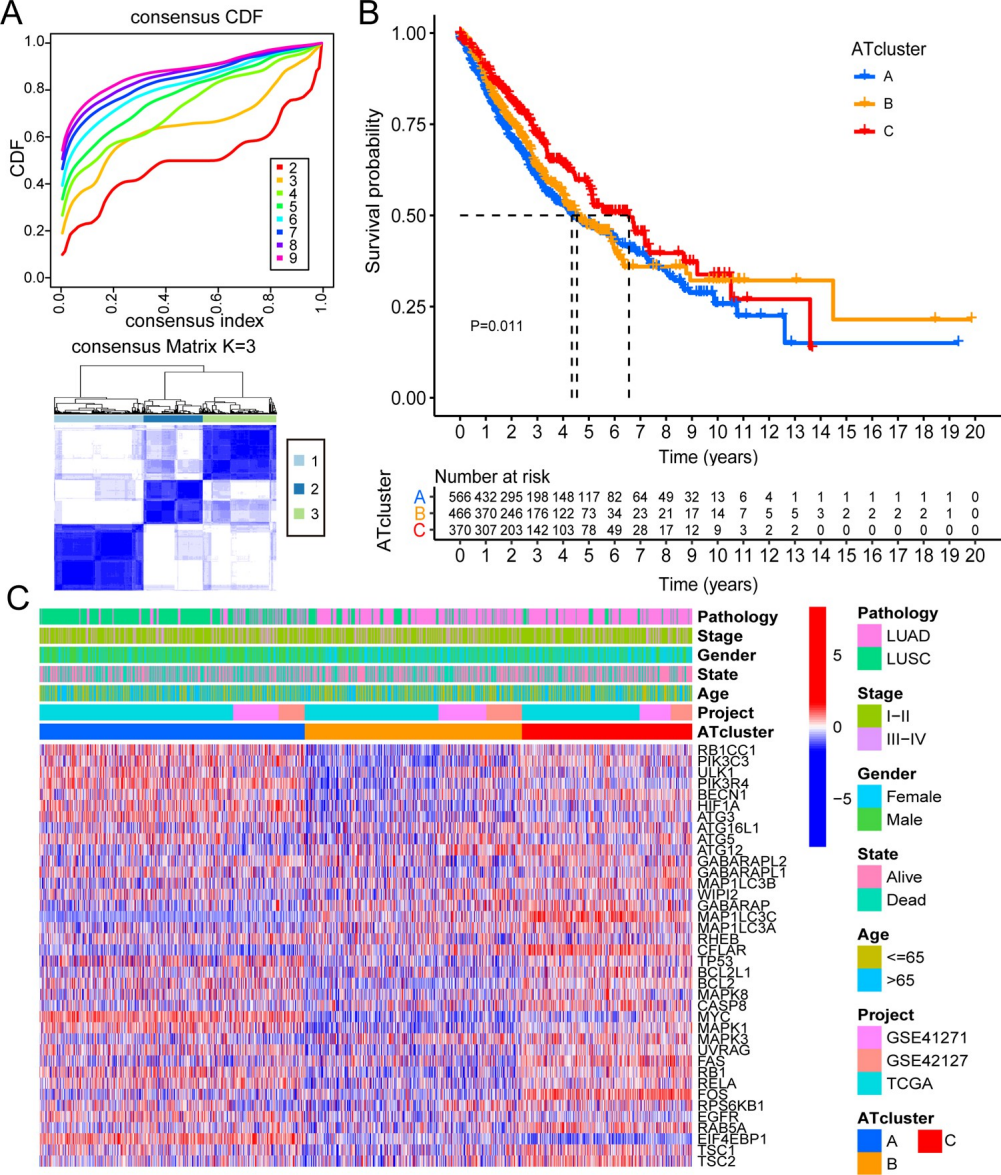
Clustering and characteristics based on ARHGs in the new combined dataset. (A) Unsupervised clustering analysis of ARHGs, inferring the optimal number of clusters by taking K as 3. (B) Survival analysis of lung cancer patients with three different degrees of autophagy in the new combined dataset. (C) Unsupervised clustering of ARHGs in the new dataset. Pathology, stage, gender, survival status, age, and source of dataset were used as patient annotations. Red represented high expression and blue represented low expression.

**Fig 4:**
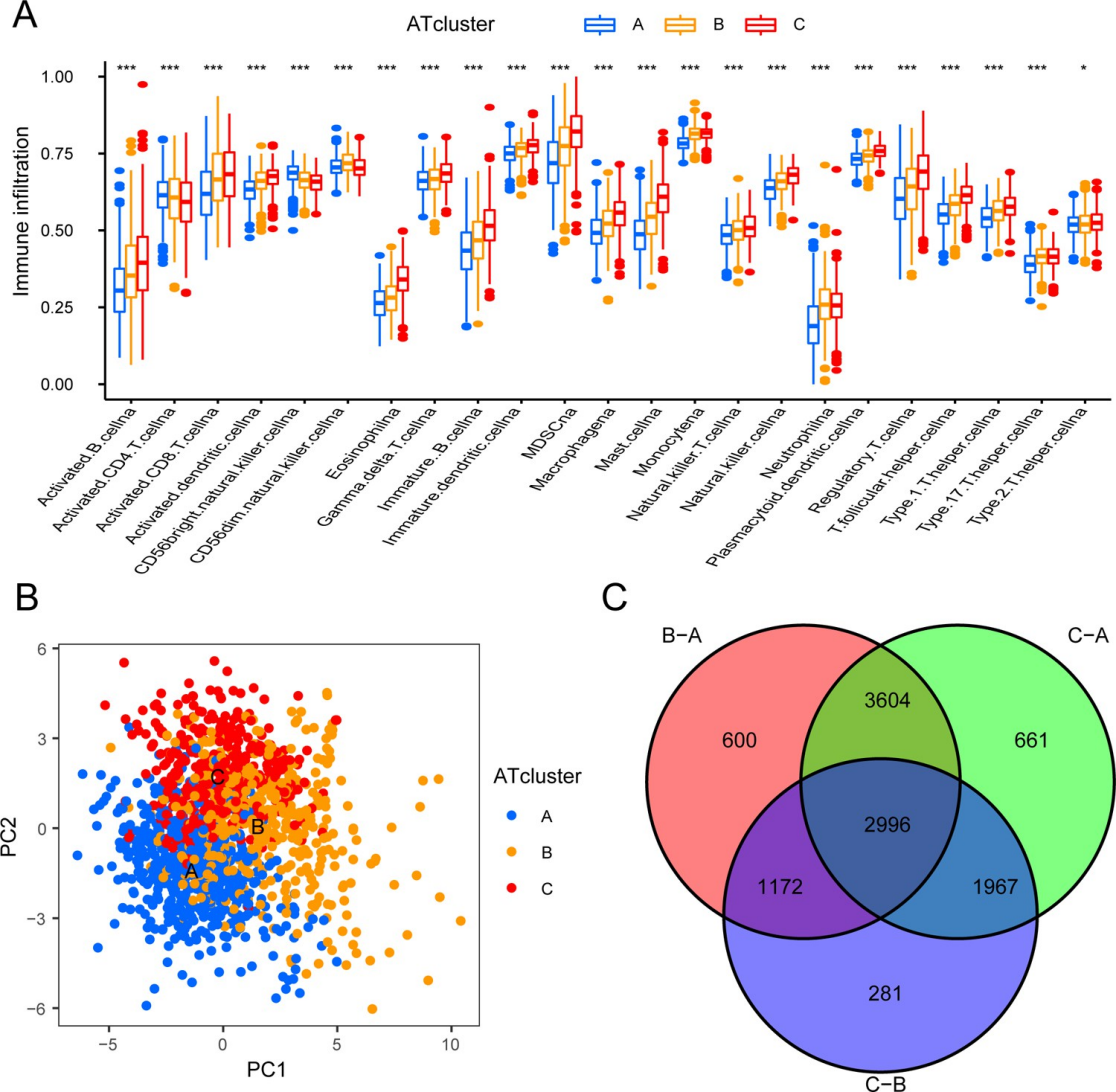
The infiltration of immune cells between ATclusters and the production of ARGs based on three ATclusters. (A) The abundance of each immune infiltrating cell in three ATclusters. The upper and lower ends of the box indicated the interquartile range of values. The line in the box represented the median value, and the points outside the box represented outliers. The asterisk represented the P-value (*P<0.05; **P<0.01; ***P<0.001). (B) The principal component analysis of the transcriptome profile under three ATclusters showed that transcriptomes under three ATclusters were significantly different. (C) 2996 autophagy-related genes shown in the venn diagram.

According to the expression of 38 ARHGs, we used unsupervised clustering method to classify tumor samples. The full name of the CDF value was cumulative distribution function value which was the integral of the probability density function. K was the number of clusters. The consistent cumulative distribution function (CDF) graph showed the cumulative distribution function when k took different values. It was used to determine when k took a value, the CDF reached an approximate maximum value, and the cluster analysis result at this time was the most reliable. That was, considering the value of k with a small slope of CDF. Finally, we found that when K was set to 3, the best CDF value was obtained. The tumor samples were split into 3 different groups (Fig 3A), and were termed ATcluster A (573 samples), ATcluster B (469 samples), ATcluster C (377 samples) respectively. There were significant differences between these three different clusters (Fig 4B). Survival analysis was performed on these three clusters, and it was found that ATcluster C had a more survival advantage (Fig 3B). It can be seen from the heat map that the expression levels of ARHGs were dissimilar between different clusters and the expression levels in ATcluster B were lower than those in the other two clusters. We also found that in male patients with LUSC, most of the deaths were concentrated in ATcluster A, rather than in ATclusters B and ATcluster C (Fig 3C) [33].

### Differences in function and immune infiltration between ATclusters

**Fig 5:**
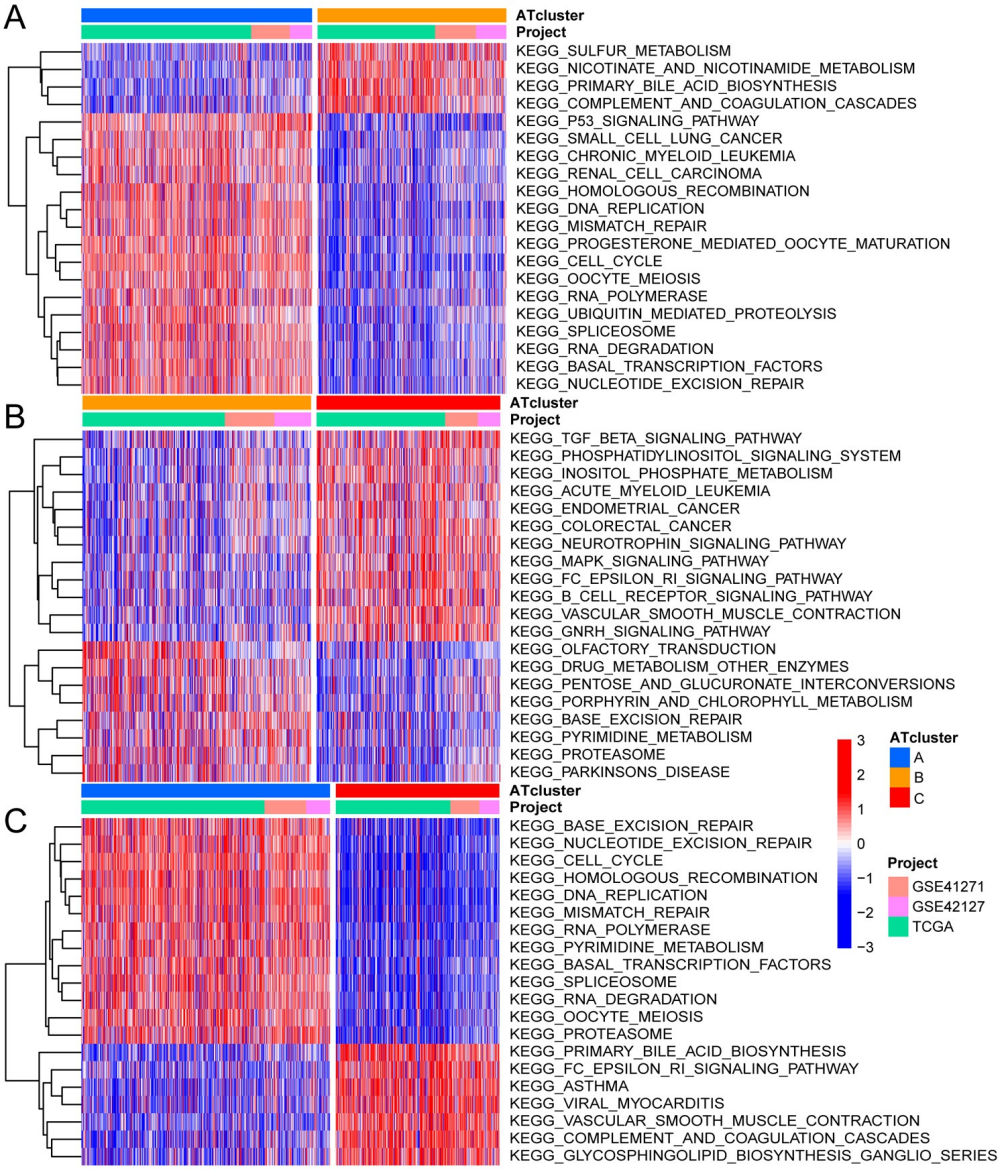
GSVA enrichment analysis among three ATclusters. (A) GSVA enrichment analysis between ATcluster A and ATcluster B, (B) ATcluster B and ATcluster C, (C) ATcluster A and ATcluster C, Which showed the changes of various biological pathways under different degrees of autophagy. The heat map showed the status of these pathways, with red representing the activation pathway and blue representing the inhibition pathway. The lung cancer cohort was used as sample annotation.

Based on the merger cohort, we used GSVA to explore the potential role of these ARHGs, and pathways with P-value less than 0.05 would be considered meaningful [34]. It could be clearly seen that the functions of three ATclusters were different. ATcluster A was actively involved in pathways related to cell replication and repair, such as DNA replication, cell cycle, mismatch repair, nucleotide excision repair and RNA degradation. While cluster C was significantly enriched in tumor suppressor and immune pathways, including TGF-β signaling pathway, Fc epsilon RI signaling pathway, B cell receptor signaling pathway, complement and coagulation cascades and phosphatidylinositol signaling system. ATcluster B mainly focused on biological metabolic pathways such as sulfur metabolism, pentose and glucuronate interconversions, porphyrin and chlorophyll metabolism and pyrimidine metabolism (Fig 5A–5C). We subsequently performed GSEA analysis on these three clusters, and found that the infiltration of immune cells between clusters was very different. ATcluster C contained abundant immune cell infiltration, ATcluster A was the least, and ATcluster B lay between the two. While activated CD4 T cell and CD56bright natural killer cell were the opposite (Fig 4A). Surprisingly, patients in ATcluster C showed a corresponding survival advantage (Fig 3B). Combined with the GSVA analysis results, we speculated that the infiltration of immune cells might play an important role in antitumor [26].

### Identification of DEGs related to autophagy and geneClusters

In order to identify potential DEGs related to autophagy, we performed a differential analysis on the merger cohort based on three ATsclusters. As showed in Fig 4C, a total of 2996 DEGs related to autophagy were selected (adjusted P-value < 0.001). To explore the potential functions of these DEGs, GO and KEGG enrichment analysis were used. We obtained a total of 1369 GO terms (P-value <0.05, q-value <0.05) and 43 KEGG pathways (P-value <0.05, q-value <0.05). Sorting according to the number of genes from high to low, we selected the top 30 GO terms which included 10 BP (biological process) terms, 10 CC (cellular component) terms and 10 MF (molecular function) terms and 30 KEGG pathways respectively. The results showed that 30 GO terms and 30 KEGG pathways mentioned above were mainly concentrated in immune response−activating cell surface receptor signaling pathway, negative regulation of immune system process, neutrophil activation involved in immune response, T cell activation, ubiquitin−like protein ligase binding, protein serine/threonine kinase activity, TNF signaling pathway, and B cell receptor signaling pathway (S2A and S2B Fig) [35].

**Fig 6:**
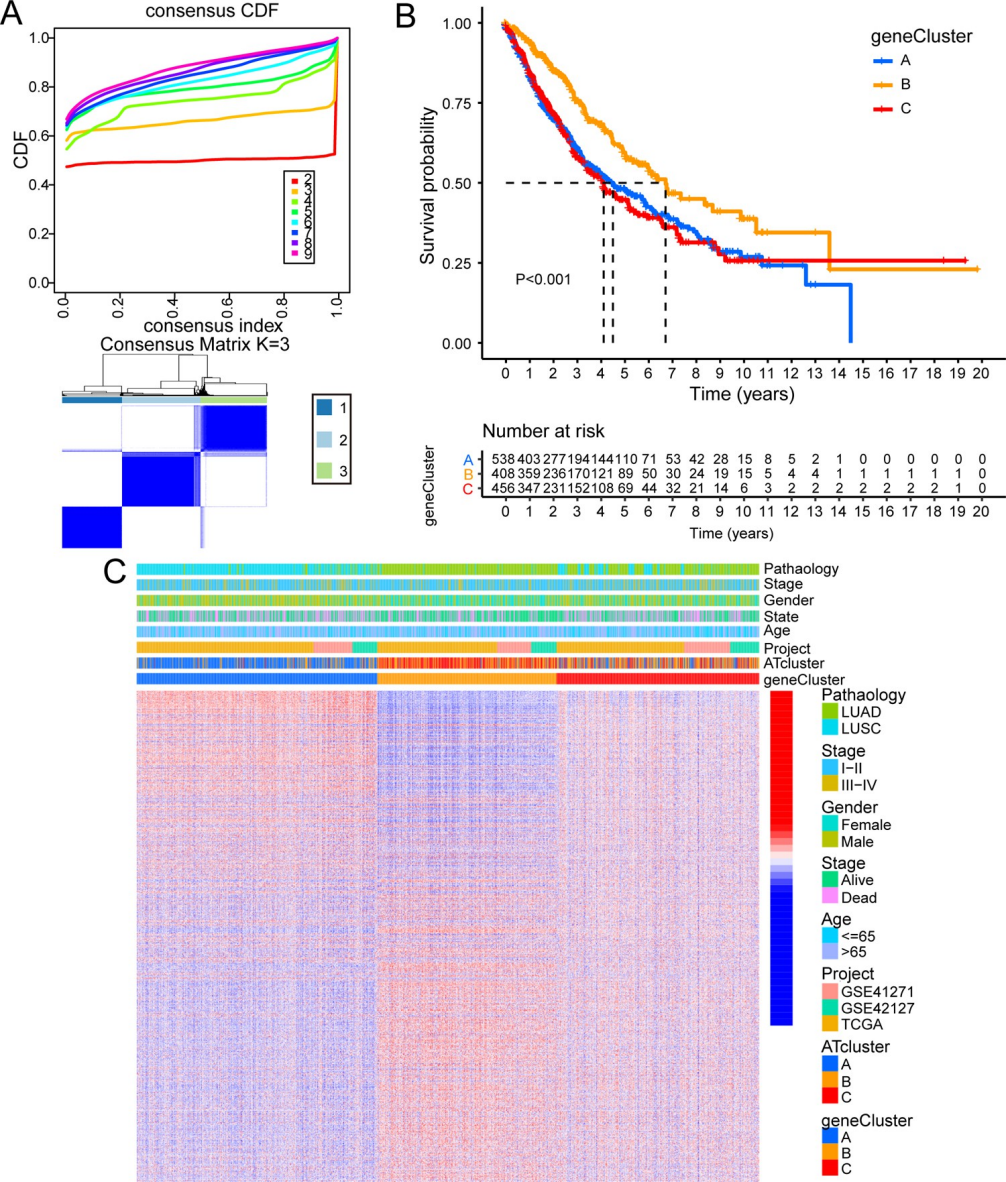
Clustering and characteristics based on ARGs which were related to prognosis in the new combined dataset. (A) Unsupervised clustering analysis of 1803 ARGs related to prognosis, inferring the optimal number of clusters by taking K as 3. (B) Survival analysis comparison among three geneclusters. (C) Unsupervised clustering of ARGs related to prognosis in the new dataset. Pathology, stage, gender, survival status, age, ATcluster and source of data set were used as patient annotations. Red represented high expression and blue represented low expression.

We then performed univariate Cox analysis on these 2996 DEGs related to autophagy, and finally identified 1803 DEGs (P-value <0.05) related to prognosis. In order to further verify the regulatory mechanism of autophagy, we performed unsupervised clustering analysis based on the 1803 genes obtained. Surprisingly, consistent with the previous ARHGs clustering, the patients were also divided into three clusters (Fig 6A). The three clusters were named geneCluster A~C. Survival analysis showed that geneCluster B had the most advantage in survival (Fig 6B). It could be found that female LUSC patients were mainly concentrated in geneCluster A (Fig 6C). Subsequently, based on three geneClusters, we performed a differential analysis of the expression levels of 38 ARHGs in merger cohort with “Limma” R package. As expected, there was a significant difference in the expression of ARHGs between the three geneClusters (S3A Fig).

### The relationship between ATscore and traits of each subtype

**Fig 7:**
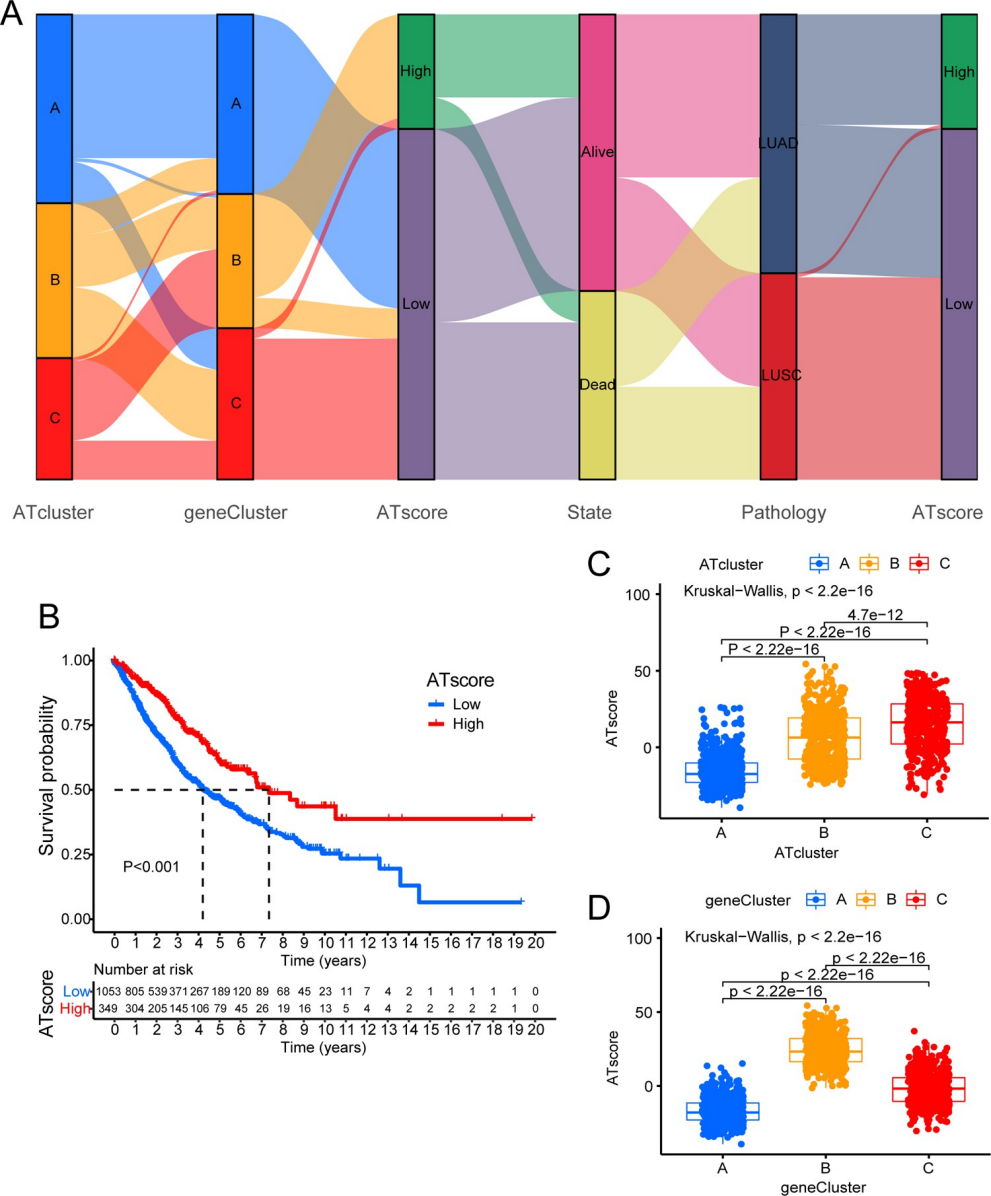
Establishment of autophagy scoring system and its related characteristics. (A) Sankey diagram showing the changes of ATcluster, genecluster, survival status, pathology and ATscore. (B) The K-M curve for the low ATscore and the high ATscore groups divided by autophagy related signature in the new combined dataset. (C) The difference of ATscore among three ATclusters. The Kruskal-Wallis test was used to compare the statistical differences between three ATclusters. (D) The difference of ATscore among three geneclusters (Kruskal-Wallis test, P<0.001).

Due to individual differences, we constructed a scoring system which was termed ATscore to quantify the degree of autophagy in each patient based on 1803 DEGs related to prognosis. Survival analysis revealed that patients with high ATscore were better than those with low ATscore in survival (Fig 7B). The attribute changes of each patient were presented in the Sankey chart (Fig 7A). We also assessed the correlation between immune cells and ATscore (S1B Fig). The Kruskal-Wallis test revealed that the ATscore was significantly different between ATclusters and geneClusters. Among ATclusters, ATcluster A had the lowest ATscore and ATcluster C had the highest score (Fig 7C). Analysis of GSVA and ssGSEA showed that ATcluster A was mainly related to the occurrence and development of tumor cells, and ATcluster C was mainly related to tumor suppression and immunity (Fig 5A–5C). Therefore, we speculated that patients with high ATscore tended to suppress tumors, while those with low ATscore tended to be subjected to tumors. Among geneClusters, geneCluster B had the highest ATscore, and geneCluster A had the lowest ATscore (Fig 7D). Combined with previous survival analysis (Figs 3B and 6B), it also could be seen that patients with higher ATscore had more advantage in survival which was consistent with the result (Fig 7B).

**Fig 8:**
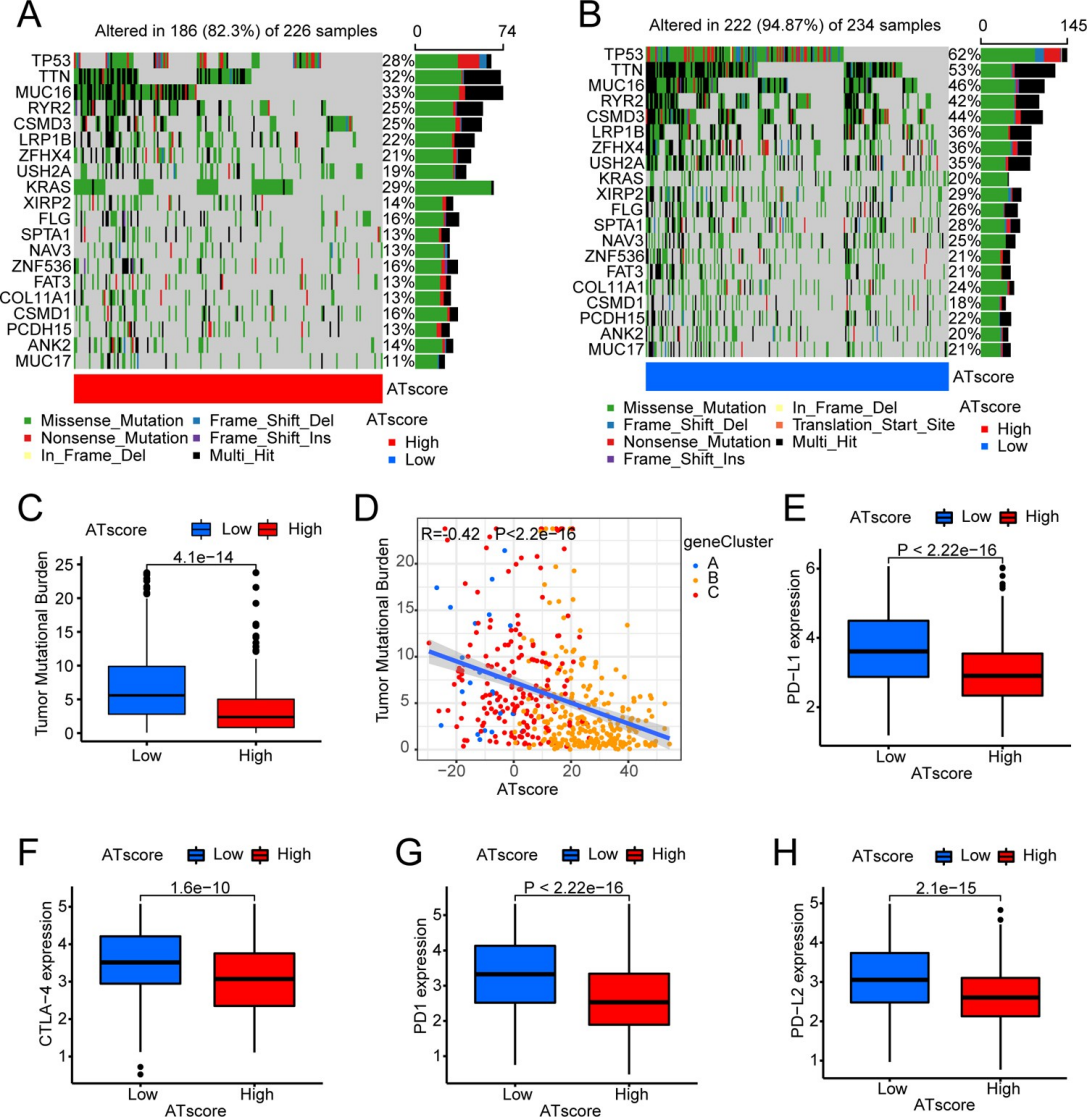
Characteristics of mutations and expression of common immune checkpoints between high and low ATscore groups in LUAD. The mutation status of the top 20 ARHGs in (A) the high autophagy score and (B) the low autophagy score groups. (C) Difference of TMB between high autophagy score group and low autophagy score groups. The thick line represented the median value, and the upper and lower parts of the box represented the interquartile range. Black dots represented outliers (Wilcoxon test, P<0.05). (D) The relationship between TMB and ATscore. The scatter plot indicated that the ATscore value was negatively correlated with TMB. Each dot represented a patient, and the color of the dot represented the source group. (E~H) The expression of common immunotherapy genes between high ATscore and low ATscore groups.

**Fig 9:**
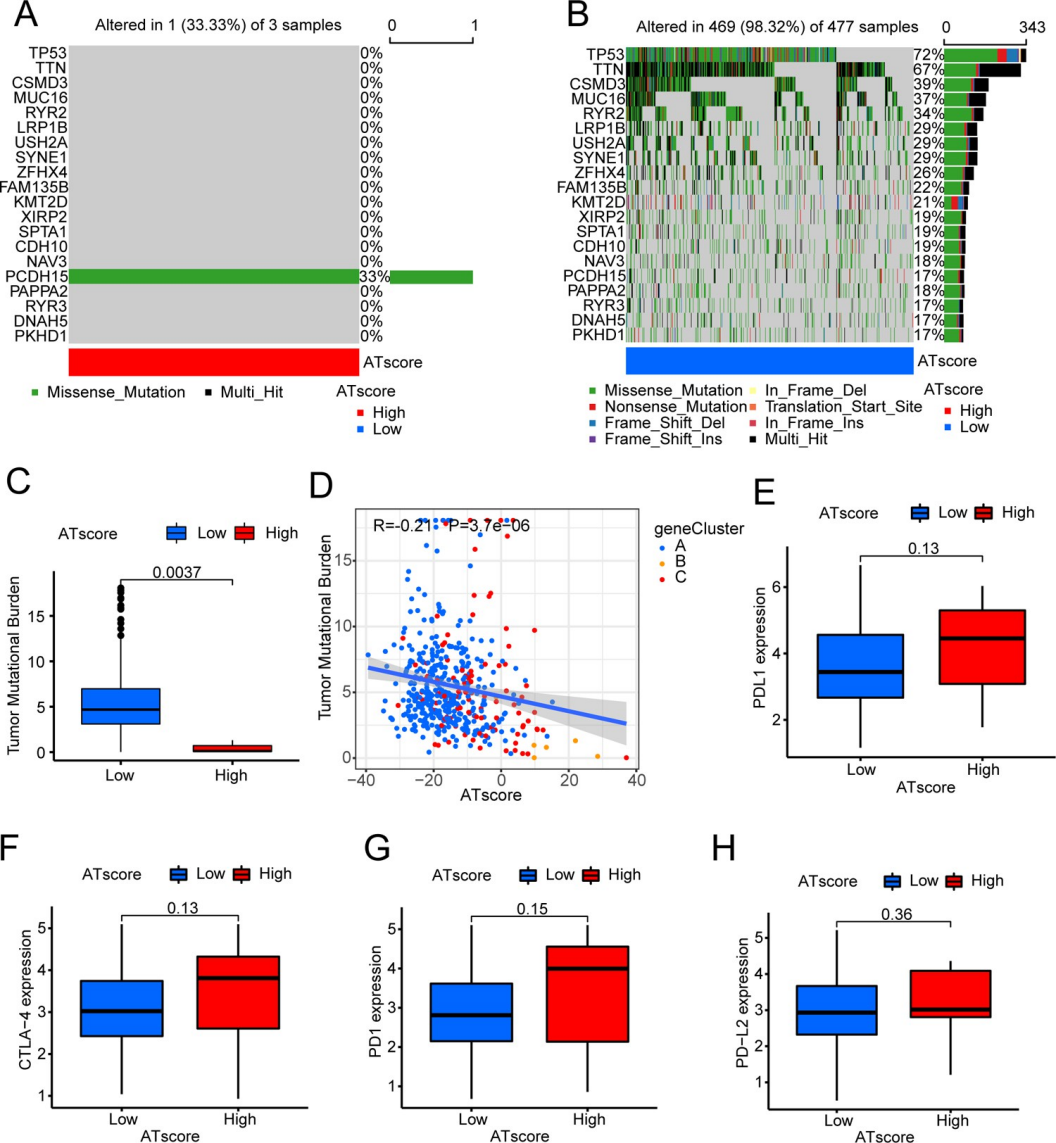
Characteristics of mutations and expression of common immune checkpoints between high and low ATscore groups in LUSC. (A) the high autophagy score and (B) the low autophagy score groups. (C) Difference of TMB between high autophagy score group and low autophagy score groups. The thick line represented the median value, and the upper and lower parts of the box represented the interquartile range. Black dots represented outliers (Wilcoxon test, P<0.05). (D) The relationship between TMB and ATscore. The scatter plot indicated that the ATscore value was negatively correlated with TMB. Each dot represented a patient, and the color of the dot represented the source group. (E~H) The expression of common immunotherapy genes between high ATscore and low ATscore groups.

For the purpose of exploring the relationship between ATscore and occurrence and development of tumor, we conducted an analysis of the relationship between TMB and ATscore in LUAD and LUSC respectively. In LUAD, through quantitative analysis of TMB, it could be seen that tumors with low ATscore exhibited high TMB (Fig 8C). There was likewise a significant negative correlation between ATscore and TMB (Fig 8D). In order to further test this relationship, we divided LUAD patients into high and low groups based on ATscore. It was found that the degree of gene mutation in the group with low ATscore was greater than that in the group with high ATscore (Fig 8A and 8B). We then analyzed LUSC patients in the same way. Surprisingly, the results were similar to LUAD (Fig 9A–9D). We also explored the impact of TMB and ATscore on survival. In LUAD, patients with high TMB and high ATscore survived longer (S3B and S3C Fig). In LUSC, although it might be affected by the number of samples, we could still see that patients with high TMB had a survival benefit (S3D and S3E Fig).

### ATscore was a prognostic biomarker

We evaluated the correlation between ATscore and clinical characteristics (age, gender, stage, pathology). It was found that there were significant differences between clinical characteristics (except age) and ATscores. Between the high-score and low-score groups, there was no difference in age distribution. Patients younger than or equal to 65 years old accounted for 45%, and those older than 65 years old accounted for 55% (S4A Fig). Quantitative analysis showed that the ATscore between patients less than or equal to 65 years old and patients older than 65 years old had no meaning (S4F Fig). The surviving female LUAD patients with stageⅠ~Ⅱwere mostly concentrated in the high ATscore group, while those who died of stage Ⅲ~Ⅳ male LUSC patients were mostly concentrated in the low ATscore group (S4B–S4E Fig). By quantitative analysis, the ATscore of surviving patients was higher than that of dead patients (S4G Fig). Using the same method to analyze gender, pathology, and stage, we found that female had higher ATscore than male (S4H Fig), patients with LUAD had higher ATscore than patients with LUSC (S4I Fig) and ATscore in patients with stage Ⅰ~Ⅱ were higher than those with stage Ⅲ~Ⅳ (S4J Fig).

In order to further test the applicability of the ATscore system, we divided patients with the same clinical trait into high ATscore group and low ATscore group, and analyzed them for survival. To our surprise, in each individual clinical trait, patients with high ATscore had better survival than patients with low ATscore (S5A–S5H Fig). Although the P-value of patients with LUSC who were less than or equal to 65 years old and stage which was Ⅲ~Ⅳ was greater than 0.05, they had a certain guiding effect on the prognosis (S5B–S5F and S5H Fig).

To explore the relationship between the prognosis of LUSC and LUAD and the ATscore, we divided patients with the same clinical characteristic into two groups based on pathology. We first performed survival analysis on them separately. Although their P-value was greater than 0.05 (except for patients with stage Ⅰ~Ⅱ), their results indicated that the prognosis of LUAD with the same clinical characteristic was better than that of LUSC (S6A–S6F Fig). As expected, through quantitative analysis, it was found that the ATscore of patients with LUAD with the same clinical characteristic was higher than that of patients with LUSC (S7A–S7F Fig), which further validated the results obtained before (S4D and S4I Fig).

Next, we conducted univariate and multivariate cox analysis of ATscore and clinical characteristics (age, gender, stage, pathology). Univariate cox analysis showed that age, stage, gender, and pathology were risk factors for patient prognosis, while ATscore was a protective factor. Results of multivariate cox analysis showed that age, stage, and ATscore could be used as independent prognostic factors for evaluating the prognosis of patients (Table 2).

**Table 2:** Univariate Cox analysis and multivariate cox analysis of ATscore.

Cox analysis	Characteristics	HR(95%CI)	P-value
Univariate Cox analysis	Age	1.333(1.125–1.580)	<0.001
	Gender	1.315(1.107–1.563)	0.002
	Stage	2.059(1.722–2.462)	<0.001
	Pathology	1.286(1.089–1.519)	0.003
	ATscore	0.990(0.986–0.994)	<0.001
Multivariate Cox analysis	Age	1.347(1.134–1.601)	<0.001
	Gender	1.141(0.954–1.366)	0.149
	Stage	2.001(1.672–2.395)	<0.001
	Pathology	0.958(0.772–1.191)	0.702
	ATscore	0.991(0.985–0.997)	0.002

Table 2 presented the results of univariate and multivariate COX analysis of clinical characteristics and the signature ATscore.

**Fig 10:**
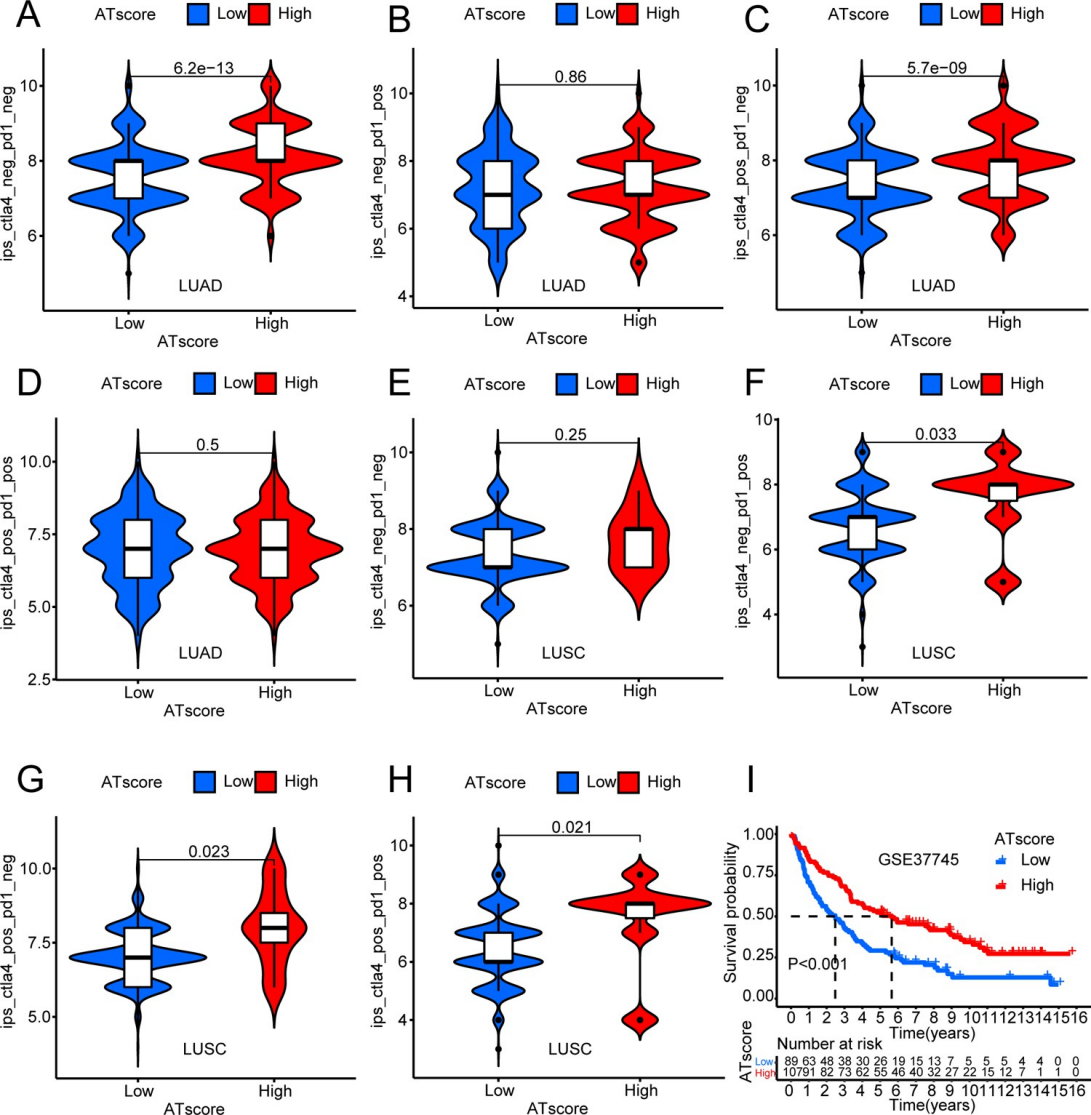
Relationship between immunophenotypic score and high and low ATscore groups. The immunophenotypic score of CTLA-4 negative and PD1 negative in high ATscore group and low ATscore group in (A) LUAD and (E) LUSC. The immunophenotypic score of CTLA-4 negative and PD1 positive in high ATscore group and low ATscore group in (B) LUAD and (F) LUSC. The immunophenotypic score of CTLA-4 positive and PD1 negative in high ATscore group and low ATscore group in (C) LUAD and (G) LUSC. The immunophenotypic score of CTLA-4 positive and PD1 positive in high ATscore group and low ATscore group in (D) LUAD and (H) LUSC. The upper and lower ends of the box indicated the interquartile range of immunophenotypic score, and the horizontal line in the box indicated the median value of expression. The points outside the box represented outliers. (I) Survival analysis of patients between high and low ATscore groups in validation set GSE37745.

From the validation of patients in GSE37745, we were surprised to find that the results were as expected—patients with high autophagy lived longer (Fig 10I).

### Treatment strategy of LUAD and LUSC based on ATscore

We assessed the expression levels of four common immune checkpoints (PD1, PD-L1, PD-L2, and CTLA-4) in LUAD and LUSC, and analyzed differences based on the patients’ ATscore. From the results, it could be seen that the immune checkpoint molecules were lowly expressed in patients with high ATscore, and were highly expressed in patients with low ATscore in LUAD (Fig 8E–8H). However, the results obtained were exactly the opposite of LUAD in LUSC, although their P-value was greater than 0.05 (Fig 9E–9H). At the same time, based on the ATscore, we analyzed the IPS score in LUAD and LUSC to predict their immunogenicity. In LUAD, IPS and IPS-CTLA4 scores were higher in patients with high ATscore (Fig 10A–10D). In LUSC, patients who were in the high ATscore group had higher IPS-PD1/PD-L1/PD-L2, IPS-CTLA4, IPS-PD1/PD-L1/PD-L2-CTLA4 scores (Fig 10E–10H). These results indicated that LUAD patients with high ATscores might respond better to CTLA4 immunotherapy, while LUSC patients with high ATscores might get a better response to CTLA4, PD1/PDL1/PDL2, CTLA4-PD1/PDL1/PDL2 immunotherapy [36].

Based on the ATscore, we also analyzed the sensitivity of several common chemotherapy drugs (Cisplatin, Gemcitabine, Paclitaxel, Vinorelbine and Methotrexate) in LUAD and LUSC. The results showed that in LUAD, patients with high ATscores were sensitive to these five chemotherapeutics (S8A–S8E Fig). In LUSC, patients with high ATscores were sensitive to cisplatin, paclitaxel, and vinorelbine (S8F–S8J Fig) [37].

## Discussion

Autophagy is a process of self-digestion, which plays a great role in maintaining cell stability. It can remove waste and harmful substances in cells, and can inhibit the occurrence of tumors. The tumor suppressor function of autophagy has been widely recognized [38]. However, recent studies have shown that autophagy has a dual effect. It can also provide nutrition for tumor cells to promote the growth of tumor cells, and allow tumor cells to evade immune surveillance which leads to drug resistance [39]. Therefore, in the process of improving the efficacy of anti-tumor drugs and reducing tumor resistance, autophagy is such as to become a key regulatory point. Research on autophagy is expected to improve the survival rate of NSCLC patients.

We conducted research on LUAD and LUSC, which accounted for the largest proportion of NSCLC. Based on the TCGA database, we conducted a preliminary exploration and found that the expression of autophagy in lung cancer was relatively active. We speculated that autophagy played a major role in the occurrence and development of lung cancer. Therefore, we conducted further research on autophagy and established a set of autophagy scoring system. We combined the autophagy score with clinical characteristics, gene expression, immune cell infiltration, and tumor mutation burden. As expected, the autophagy score was significantly correlated with immune cell infiltration, tumor mutation burden, gender, status, pathology, and staging. Survival analysis showed that patients with high autophagy score had an advantage in survival. In order to reduce the interference of other factors, we analyzed patients with the same clinical traits. Surprisingly, the conclusion still holed. Univariate and multivariate COX analysis suggested that autophagy score could be an independent prognostic factor and a protective factor for patient prognosis.

In preliminary exploration of the role of autophagy in LUAD and LUSC, mutation analysis showed that the mutation frequency of TP53 was the highest. And we were surprised to find that the expression of key autophagy genes was different between the TP53 mutant group and the TP53 wild group. In the subsequent high autophagy score group and low autophagy score group, the mutation frequency of TP53 also appeared to be the highest. As we all know, TP53 is a tumor suppressor, and the inactivation of TP53 function is a common feature of human tumors. TP53 is also involved in complex biological processes such as cell DNA repair, apoptosis, and aging [40,41]. Therefore, we could speculate that the mutation of TP53 occupied a very influential position in the development of lung cancer. Among ARGs, TP53 was likely to become an immune regulatory point that improved the survival rate of lung cancer patients and reduced drug resistance.

In the process of analyzing ATclusters, we divided the samples into ATcluster A~C according to the expression of ARHGs. Survival analysis showed that patients in ATcluster C had a longer survival time, followed by patients in ATcluster B, and patients in ATcluster A had shorter survival time. GSVA analysis suggested that ATcluster A was mainly focused on cell replication and repair, ATcluster C was mainly focused on tumor suppression and immunity, and ATcluster B was somewhere in between. Coincidentally, ATcluster A had the lowest autophagy score, ATcluster C had the highest score, and ATcluster B was in between. Combining the relationship between tumor mutation burden and autophagy score, it could be seen that the higher the autophagy score, the higher the probability of tumor mutation, and patients showed anti-tumor and immune processes, and the longer the survival time. On the contrary, the less the probability of tumor mutation, the performance was tumor cell proliferation and repair, and the survival time was shorter. A series of analyses on geneclusters further confirmed this idea.

We compared LUAD patients and LUSC patients with the same clinical traits. From the survival analysis, it could be seen that the survival time of LUAD patients was longer than that of LUSC patients, and the autophagy score of LUAD was higher than that of LUSC. We could conclude that the lower the autophagy score, the less conducive to survival, and the higher the degree of malignancy of the tumor. In LUAD, although the expression level of CTLA-4 in patients with high autophagy score was higher than that in patients with low autophagy score, immunogenicity analysis suggested that the immunogenicity of CTLA-4 in patients with high autophagy score was higher than that of patients with low autophagy score. It can be speculated that LUAD patients with high autophagy scores were more suitable for CTLA-4 immunotherapy. In LUSC, the expression level of immune checkpoints in patients with high autophagy scores was greater than that in patients with low autophagy scores, and the immunogenicity of CTLA-4/PD1 in patients with high autophagy scores was higher than that of patients with low autophagy scores too. It could be seen that LUSC patients with high autophagy score were suitable for CTLA-4, PD1 or combined immunotherapy. Sensitivity analysis of common chemotherapy drugs proved that patients with high autophagy score were more sensitive. The above analysis confirmed that the prognosis of patients with high autophagy score was better from another angle [42]. Survival analysis of patients between high and low ATscore in GSE37745 further confirmed this model.

Although the autophagy score has a good predictive and prognostic guiding role for LUAD and LUSC patients, there are still some limitations that needed to be resolved. First of all, these samples are collected from public databases, and there may be selection bias. Secondly, the transcription of the genes in the database comes from tumor tissues (including the tumor itself and the tumor microenvironment), so it is impossible to distinguish where the signature (ARG and ARHG) in this research mainly comes from. Thirdly, infiltration of immune cells, tumor mutational burden, effects of immunotherapy and commonly used chemotherapeutics are only inferred by computer calculations, and clinical verification is lacking. Studies have shown that autophagy has its duality in lung cancer. It can inhibit cancer and promote cancer. In this article, the autophagy score only roughly reflects the side of autophagy inhibiting cancer, and does not reflect the side of excessive autophagy promoting cancer. Therefore, further experiments and clinical data are needed to confirm and perfect this finding [43].

## Supporting information

S1 Fig The relationship between ARHGs. (A) The relationship between ARHGs in LUAD and LUSC in TCGA database. Red represented positive correlation, and blue represented negative correlation. The relationship coefficients were shown in the box. (B) Correlation between ATscore and known immune cells. Blue represented negative correlation while red represented positive correlation. The asterisk indicated that the P value was less than 0.05.
(TIF)

S2 Fig Functional and biological characteristics of ARGs based on three ATclusters. Functional annotation for 2996 autophagy-related genes by using the (A) GO and (B) KEGG enrichment analysis. The length of the bar represented the number of enriched genes, and the color of the bar reflected the q-value.
(TIF)

S3 Fig The expression of ARHGs among geneclusters and the relationship between TMB, ATscore and prognosis. (A) Differences in the expression levels of ARHGs among three geneclusters. The upper and lower ends of the box indicated the interquartile range of values. The line in the box represented the median value, and the points outside the box represented outliers. The asterisk represented the P-value (*P<0.05; **P<0.01; ***P<0.001). Survival analyses for subgroup patients divided by TMB and ATscore in (B~C) LUAD and (D~E) LUSC.
(TIF)

S4 Fig Relationships between ATscore and clinical features in the new combined dataset. (A) The proportion of age: patients older than 65 and patients younger than 65 or equal to 65. (B) The proportion of survival outcome: alive and dead. (C) The proportion of gender: female and male. (D) The proportion of pathological type: LUAD and LUSC. (E) The proportion of tumor stage: stage I-II and stage III-IV. The autophagy signature in cohorts stratified by lung cancer patients (F) older than 65 and younger than or equal to 65, (G) patients who were dead and patients who were alive, (H) female patients and male patients, (I) LUAD patients and LUSC patients, (J) patients whose tumor stage were stage I-II and patients whose tumor stage were stage III-IV. The box plot showed that ATscore was not statistically significant in age.
(TIF)

S5 Fig Survival differences between high and low ATscore groups with different clinical characteristics. The relationship between OS and autophagy signature in (A) patients over 65 years old, (B) patients younger than or equal to 65, (C) female patients, (D) male patients, (E) LUAD patients, (F) LUSC patients, (G) patients in stage I-II, (H) patients in stage III-IV. The K-M plots showed that the ATscore in patients younger than or equal to 65 and whose tumor pathological were LUSC and whose tumor stage were III-IV was not statistically significant.
(TIF)

S6 Fig Survival differences between LUAD and LUSC with different clinical characteristics. Survival analysis of LUAD and LUSC patients with the same clinical features: (A) age < = 65, (B) age >65, (C) male, (D) female, (E) stage I-II, (F) stage III-IV. K-M curves showed that it was statistically significant when the patient is in stage I-II. Although the remaining P values were greater than 0.05, it could be inferred from the figure that the prognosis of patients with LUAD with the same clinical characteristics was better than that of patients with LUSC.
(TIF)

S7 Fig Survival differences between LUAD and LUSC with different clinical characteristics. Survival analysis of LUAD and LUSC patients with the same clinical features: (A) age < = 65, (B) age >65, (C) male, (D) female, (E) stage I-II, (F) stage III-IV. K-M curves showed that it was statistically significant when the patient is in stage I-II. Although the remaining P values were greater than 0.05, it could be inferred from the figure that the prognosis of patients with LUAD with the same clinical characteristics was better than that of patients with LUSC.
(TIF)

S8 Fig Relationships between autophagy signature and sensitivity of common chemotherapeutic drugs. The drug sensitivity of (A, F) cisplatin, (B, G) gemcitabine, (C, H) methotrexate, (D, I) paclitaxel and (E, J) vinorelbine in high and low ATscore groups in LUAD and LUSC.
(TIF)

S1 Table Raw data obtained in this research. (A) Scores for each item of ARHGs in the PPI network. (B) Correlation coefficient between ARHGs in the TCGA cohort. (C) The changes of ATcluster, gene cluster, ATscore and ATscoregroup in the merger cohort. (D) The original result of GSVA on the merger cohort. (E) The expression of immune cells in the merger cohort based on the ssGSEA. (F) Functional annotation for 2996 DEGs (Gene Ontology). (G) Functional annotation for 2996 DEGs (kyoto encyclopedia of genes and genomes). (H) Correlation coefficient between ATscore and immune cells. (I) The ATcluster, ATscore and ATscoregroup in the validation cohort GSE37745.
(XLSX)

## Abbreviations

LUAD: lung adenocarcinoma
LUSC: lung squamous cell carcinoma
NSCLC: non-small cell lung cancer
ARGs: autophagy-related genes
ARHGs: autophagy-related hub genes
SCLC: small cell lung cancer
TCGA: the cancer genome atlas
GEO: gene expression omnibus
CNV: copy number variation
UCSC Xena: university of California santa cruz
FPKM: fragments per kilobase million
TPM: transcripts per million
OS: overall survival time
GSVA: Gene set variation analysis
ssGSEA: Single-sample gene set enrichment analysis
DEGs: differential expressed genes
GO: gene ontology
KEGG: kyoto encyclopedia of genes and genomes
ATscore: autophagy score
PCA: principal component analysis
IPS: immunophenotypic score
IC50: half-maximal inhibitory concentration
KM: kaplan-meier

## References

[1] Mihailidis V, Anevlavis S, Karpathiou G, Kouliatsis G, Tzouvelekis A, Zarogoulidis P, et al. Lung function changes after chemoradiation therapy in patients with lung cancer treated by three usual platinum combinations. J Thorac Dis. 2018;10(9):5435–42. doi: 10.21037/jtd.2018.08.139 ; PubMed Central PMCID: PMC6196207. 30416792 PMC6196207

[2] Nasim F, Sabath BF, Eapen GA. Lung Cancer. Med Clin North Am. 2019;103(3):463–73. doi: 10.1016/j.mcna.2018.12.006 . 30955514

[3] MacDonagh L, Gray SG, Breen E, Cuffe S, Finn SP, O’Byrne KJ, et al. BBI608 inhibits cancer stemness and reverses cisplatin resistance in NSCLC. Cancer Lett. 2018;428:117–26. doi: 10.1016/j.canlet.2018.04.008 . 29653268

[4] Li S, Yang H, Zhao M, Gong L, Wang Y, Lv Z, et al. Demethylation of HACE1 gene promoter by propofol promotes autophagy of human A549 cells. Oncol Lett. 2020;20(6):280. doi: 10.3892/ol.2020.12143 ; PubMed Central PMCID: PMC7520799. 33014158 PMC7520799

[5] Schaeffer V, Lavenir I, Ozcelik S, Tolnay M, Winkler DT, Goedert M. Stimulation of autophagy reduces neurodegeneration in a mouse model of human tauopathy. Brain. 2012;135(Pt 7):2169–77. doi: 10.1093/brain/aws143 ; PubMed Central PMCID: PMC3381726. 22689910 PMC3381726

[6] Mizushima N. Autophagy: process and function. Genes Dev. 2007;21(22):2861–73. doi: 10.1101/gad.1599207 . 18006683

[7] Levine B. Cell biology: autophagy and cancer. Nature. 2007;446(7137):745–7. doi: 10.1038/446745a . 17429391

[8] Xie K, Liang C, Li Q, Yan C, Wang C, Gu Y, et al. Role of ATG10 expression quantitative trait loci in non-small cell lung cancer survival. Int J Cancer. 2016;139(7):1564–73. doi: 10.1002/ijc.30205 . 27225307

[9] Cai J, Li R, Xu X, Zhang L, Lian R, Fang L, et al. CK1alpha suppresses lung tumour growth by stabilizing PTEN and inducing autophagy. Nat Cell Biol. 2018;20(4):465–78. doi: 10.1038/s41556-018-0065-8 . 29593330

[10] Ma F, Ding MG, Lei YY, Luo LH, Jiang S, Feng YH, et al. SKIL facilitates tumorigenesis and immune escape of NSCLC via upregulating TAZ/autophagy axis. Cell Death Dis. 2020;11(12):1028. doi: 10.1038/s41419-020-03200-7 ; PubMed Central PMCID: PMC7710697. 33268765 PMC7710697

[11] Zhang X, Shi X, Zhao H, Jia X, Yang Y. Identification and Validation of a Tumor Microenvironment-Related Gene Signature for Prognostic Prediction in Advanced-Stage Non-Small-Cell Lung Cancer. Biomed Res Int. 2021;2021:8864436. doi: 10.1155/2021/8864436 ; PubMed Central PMCID: PMC8028741. 33860055 PMC8028741

[12] Yang D, Ma Y, Zhao P, Ma J, He C. Systematic screening of protein-coding gene expression identified HMMR as a potential independent indicator of unfavorable survival in patients with papillary muscle-invasive bladder cancer. Biomed Pharmacother. 2019;120:109433. doi: 10.1016/j.biopha.2019.109433 . 31568988

[13] Zhang X, Gao F, Li N, Zhang J, Dai L, Yang H. Peroxiredoxins and Immune Infiltrations in Colon Adenocarcinoma: Their Negative Correlations and Clinical Significances, an In Silico Analysis. J Cancer. 2020;11(11):3124–43. doi: 10.7150/jca.38057 ; PubMed Central PMCID: PMC7097948. 32231717 PMC7097948

[14] Chen S, Yang D, Lei C, Li Y, Sun X, Chen M, et al. Identification of crucial genes in abdominal aortic aneurysm by WGCNA. PeerJ. 2019;7:e7873. doi: 10.7717/peerj.7873 ; PubMed Central PMCID: PMC6788446. 31608184 PMC6788446

[15] Wang Q, Wang H, Jing Q, Yang Y, Xue D, Hao C, et al. Regulation of Pancreatic Fibrosis by Acinar Cell-Derived Exosomal miR-130a-3p via Targeting of Stellate Cell PPAR-gamma. J Inflamm Res. 2021;14:461–77. doi: 10.2147/JIR.S299298 ; PubMed Central PMCID: PMC7917364. 33658824 PMC7917364

[16] Sun G, Sun K, Shen C. Human nuclear receptors (NRs) genes have prognostic significance in hepatocellular carcinoma patients. World J Surg Oncol. 2021;19(1):137. doi: 10.1186/s12957-021-02246-x ; PubMed Central PMCID: PMC8091722. 33941198 PMC8091722

[17] Li M, Chen Z, Jiang T, Yang X, Du Y, Liang J, et al. Circadian rhythm-associated clinical relevance and Tumor Microenvironment of Non-small Cell Lung Cancer. J Cancer. 2021;12(9):2582–97. doi: 10.7150/jca.52454 ; PubMed Central PMCID: PMC8040717. 33854619 PMC8040717

[18] Zhang LH, Li LQ, Zhan YH, Zhu ZW, Zhang XP. Identification of an IRGP Signature to Predict Prognosis and Immunotherapeutic Efficiency in Bladder Cancer. Front Mol Biosci. 2021;8:607090. doi: 10.3389/fmolb.2021.607090 ; PubMed Central PMCID: PMC8082411. 33937319 PMC8082411

[19] Charoentong P, Finotello F, Angelova M, Mayer C, Efremova M, Rieder D, et al. Pan-cancer Immunogenomic Analyses Reveal Genotype-Immunophenotype Relationships and Predictors of Response to Checkpoint Blockade. Cell Rep. 2017;18(1):248–62. doi: 10.1016/j.celrep.2016.12.019 . 28052254

[20] Lu M, Fan X, Liao W, Li Y, Ma L, Yuan M, et al. Identification of significant genes as prognostic markers and potential tumor suppressors in lung adenocarcinoma via bioinformatical analysis. BMC Cancer. 2021;21(1):616. doi: 10.1186/s12885-021-08308-3 ; PubMed Central PMCID: PMC8157630. 34039311 PMC8157630

[21] Deng ZM, Dai FF, Zhou Q, Cheng YX. Hsa_circ_0000301 facilitates the progression of cervical cancer by targeting miR-1228-3p/IRF4 Axis. BMC Cancer. 2021;21(1):583. doi: 10.1186/s12885-021-08331-4 ; PubMed Central PMCID: PMC8140416. 34020619 PMC8140416

[22] Zhao T, Zhang Y, Ma X, Wei L, Hou Y, Sun R, et al. Elevated expression of LPCAT1 predicts a poor prognosis and is correlated with the tumour microenvironment in endometrial cancer. Cancer Cell Int. 2021;21(1):269. doi: 10.1186/s12935-021-01965-1 ; PubMed Central PMCID: PMC8139085. 34016103 PMC8139085

[23] Liu D, Yang X, Wu X. Tumor Immune Microenvironment Characterization Identifies Prognosis and Immunotherapy-Related Gene Signatures in Melanoma. Front Immunol. 2021;12:663495. doi: 10.3389/fimmu.2021.663495 ; PubMed Central PMCID: PMC8134682. 34025664 PMC8134682

[24] Sotiriou C, Wirapati P, Loi S, Harris A, Fox S, Smeds J, et al. Gene expression profiling in breast cancer: understanding the molecular basis of histologic grade to improve prognosis. J Natl Cancer Inst. 2006;98(4):262–72. doi: 10.1093/jnci/djj052 . 16478745

[25] Liu J, Wu Z, Wang Y, Nie S, Sun R, Yang J, et al. A prognostic signature based on immune-related genes for cervical squamous cell carcinoma and endocervical adenocarcinoma. Int Immunopharmacol. 2020;88:106884. doi: 10.1016/j.intimp.2020.106884 . 32795900

[26] Song C, Guo Z, Yu D, Wang Y, Wang Q, Dong Z, et al. A Prognostic Nomogram Combining Immune-Related Gene Signature and Clinical Factors Predicts Survival in Patients With Lung Adenocarcinoma. Front Oncol. 2020;10:1300. doi: 10.3389/fonc.2020.01300 ; PubMed Central PMCID: PMC7424034. 32850406 PMC7424034

[27] Wang P, Fu Y, Chen Y, Li Q, Hong Y, Liu T, et al. Nomogram Personalizes and Visualizes the Overall Survival of Patients with Triple-Negative Breast Cancer Based on the Immune Genome. Biomed Res Int. 2020;2020:4029062. doi: 10.1155/2020/4029062 ; PubMed Central PMCID: PMC7709499. 33299869 PMC7709499

[28] An J, Lai J, Sajjanhar A, Batra J, Wang C, Nelson CC. J-Circos: an interactive Circos plotter. Bioinformatics. 2015;31(9):1463–5. doi: 10.1093/bioinformatics/btu842 . 25540184

[29] Chen Y, Miao S, Zhao W. Identification and validation of significant gene mutations to predict clinical benefit of immune checkpoint inhibitors in lung adenocarcinoma. Am J Transl Res. 2021;13(3):1051–63. ; PubMed Central PMCID: PMC8014424. 33841639 PMC8014424

[30] La Fleur L, Falk-Sorqvist E, Smeds P, Berglund A, Sundstrom M, Mattsson JS, et al. Mutation patterns in a population-based non-small cell lung cancer cohort and prognostic impact of concomitant mutations in KRAS and TP53 or STK11. Lung Cancer. 2019;130:50–8. doi: 10.1016/j.lungcan.2019.01.003 . 30885352

[31] Liu G, Pei F, Yang F, Li L, Amin AD, Liu S, et al. Role of Autophagy and Apoptosis in Non-Small-Cell Lung Cancer. Int J Mol Sci. 2017;18(2). doi: 10.3390/ijms18020367 ; PubMed Central PMCID: PMC5343902. 28208579 PMC5343902

[32] Liu Q, Wang JJ, Pan YC, Meng LF, Zhan X, Zheng QF. [Expression of autophagy-related genes Beclin1 and MAPLC3 in non-small cell lung cancer]. Ai Zheng. 2008;27(1):25–9. . 18184459

[33] Qin N, Ma Z, Wang C, Zhang E, Li Y, Huang M, et al. Comprehensive characterization of functional eRNAs in lung adenocarcinoma reveals novel regulators and a prognosis-related molecular subtype. Theranostics. 2020;10(24):11264–77. doi: 10.7150/thno.47039 ; PubMed Central PMCID: PMC7532687. 33042282 PMC7532687

[34] Xiao G, Zhang X, Zhang X, Chen Y, Xia Z, Cao H, et al. Aging-related genes are potential prognostic biomarkers for patients with gliomas. Aging (Albany NY). 2021;13(9):13239–63. doi: 10.18632/aging.203008 ; PubMed Central PMCID: PMC8148480. 33946049 PMC8148480

[35] Yu Y, Tian X. Analysis of genes associated with prognosis of lung adenocarcinoma based on GEO and TCGA databases. Medicine (Baltimore). 2020;99(19):e20183. doi: 10.1097/MD.0000000000020183 ; PubMed Central PMCID: PMC7220259. 32384511 PMC7220259

[36] Yi M, Li A, Zhou L, Chu Q, Luo S, Wu K. Immune signature-based risk stratification and prediction of immune checkpoint inhibitor’s efficacy for lung adenocarcinoma. Cancer Immunol Immunother. 2021;70(6):1705–19. doi: 10.1007/s00262-020-02817-z ; PubMed Central PMCID: PMC8139885. 33386920 PMC8139885

[37] Huang Y, Lei L, Liu Y. Propofol Improves Sensitivity of Lung Cancer Cells to Cisplatin and Its Mechanism. Med Sci Monit. 2020;26:e919786. doi: 10.12659/MSM.919786 ; PubMed Central PMCID: PMC7142322. 32225124 PMC7142322

[38] Mah LY, Ryan KM. Autophagy and cancer. Cold Spring Harb Perspect Biol. 2012;4(1):a008821. doi: 10.1101/cshperspect.a008821 ; PubMed Central PMCID: PMC3249624. 22166310 PMC3249624

[39] Perera RM, Stoykova S, Nicolay BN, Ross KN, Fitamant J, Boukhali M, et al. Transcriptional control of autophagy-lysosome function drives pancreatic cancer metabolism. Nature. 2015;524(7565):361–5. doi: 10.1038/nature14587 ; PubMed Central PMCID: PMC5086585. 26168401 PMC5086585

[40] Mogi A, Kuwano H. TP53 mutations in nonsmall cell lung cancer. J Biomed Biotechnol. 2011;2011:583929. doi: 10.1155/2011/583929 ; PubMed Central PMCID: PMC3035360. 21331359 PMC3035360

[41] Jin S, Levine AJ. The p53 functional circuit. J Cell Sci. 2001;114(Pt 23):4139–40. doi: 10.1242/jcs.114.23.4139 . 11739646

[42] Buchbinder EI, Desai A. CTLA-4 and PD-1 Pathways: Similarities, Differences, and Implications of Their Inhibition. Am J Clin Oncol. 2016;39(1):98–106. doi: 10.1097/COC.0000000000000239 ; PubMed Central PMCID: PMC4892769. 26558876 PMC4892769

[43] Zhu J, Wang M, Hu D. Development of an autophagy-related gene prognostic signature in lung adenocarcinoma and lung squamous cell carcinoma. PeerJ. 2020;8:e8288. doi: 10.7717/peerj.8288 ; PubMed Central PMCID: PMC6953332. 31938577 PMC6953332

