# Supplementary figures and images for: Autophagy characteristics and establishment of autophagy prognostic models in lung adenocarcinoma and lung squamous cell carcinoma
Source: PLoS One. 2022 Mar 25;17(3):e0266070. doi: 10.1371/journal.pone.0266070 (PMC8956171; doi:10.1371/journal.pone.0266070)

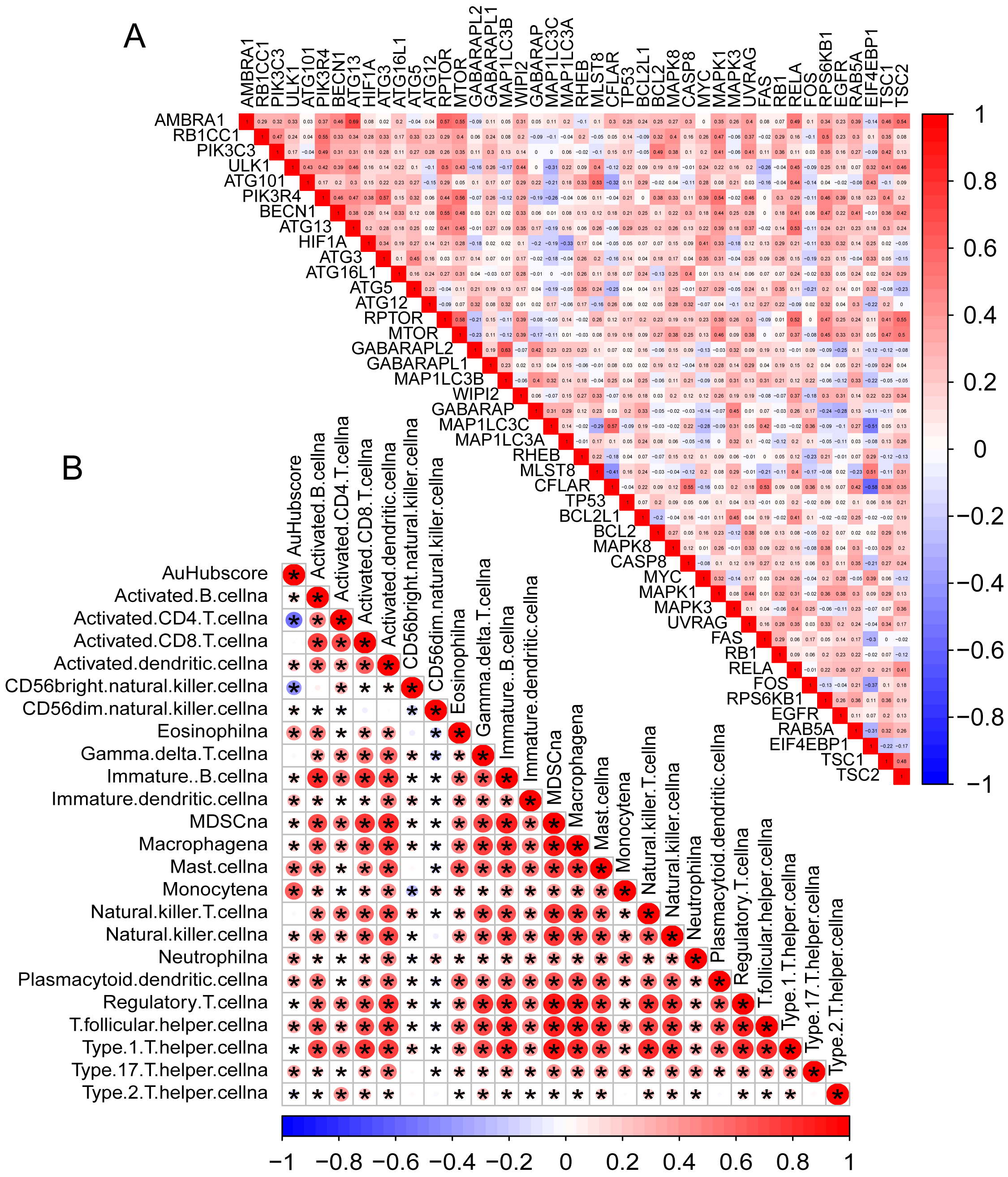

Supplement: S1 Fig — (A) The relationship between ARHGs in LUAD and LUSC in TCGA database. Red represented positive correlation, and blue represented negative correlation. The relationship coefficients were shown in the box. (B) Correlation between ATscore and known immune cells. Blue represented negative correlation while red represented positive correlation. The asterisk indicated that the P value was less than 0.05. (TIF) [file pone.0266070.s001.tif]

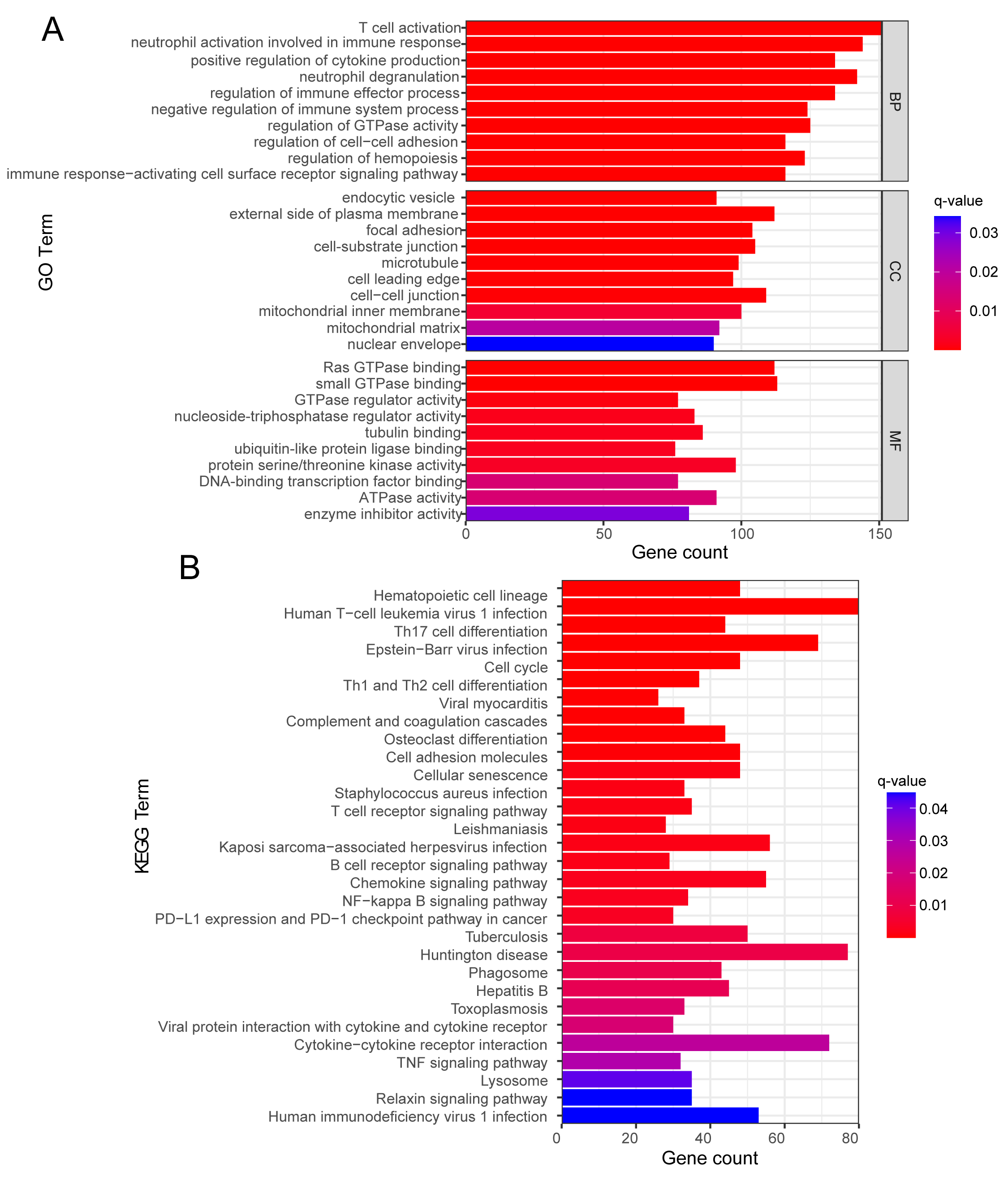

Supplement: S2 Fig — Functional annotation for 2996 autophagy-related genes by using the (A) GO and (B) KEGG enrichment analysis. The length of the bar represented the number of enriched genes, and the color of the bar reflected the q-value. (TIF) [file pone.0266070.s002.tif]

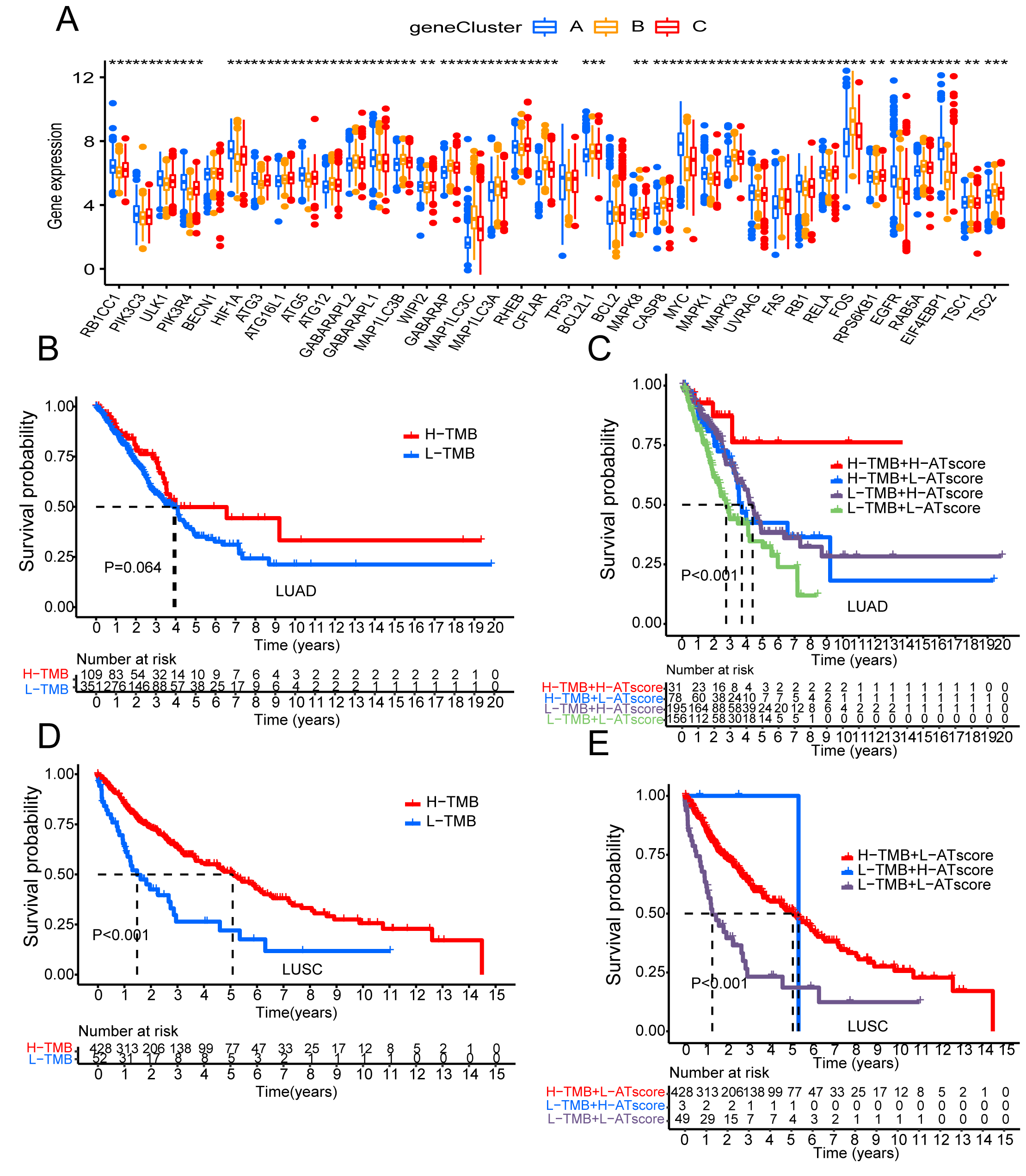

Supplement: S3 Fig — (A) Differences in the expression levels of ARHGs among three geneclusters. The upper and lower ends of the box indicated the interquartile range of values. The line in the box represented the median value, and the points outside the box represented outliers. The asterisk represented the P-value (*P<0.05; **P<0.01; ***P<0.001). Survival analyses for subgroup patients divided by TMB and ATscore in (B~C) LUAD and (D~E) LUSC. (TIF) [file pone.0266070.s003.tif]

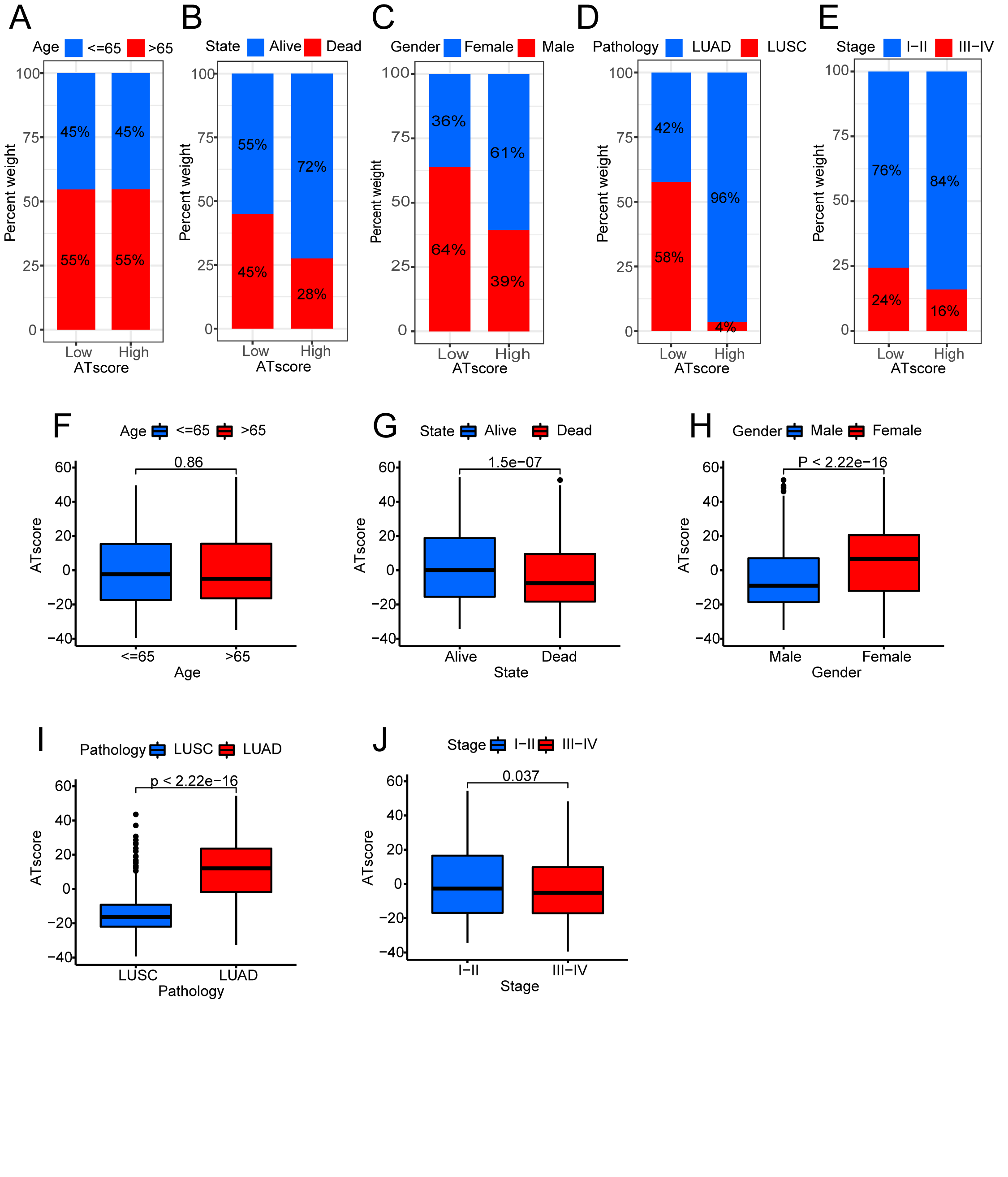

Supplement: S4 Fig — (A) The proportion of age: patients older than 65 and patients younger than 65 or equal to 65. (B) The proportion of survival outcome: alive and dead. (C) The proportion of gender: female and male. (D) The proportion of pathological type: LUAD and LUSC. (E) The proportion of tumor stage: stage I-II and stage III-IV. The autophagy signature in cohorts stratified by lung cancer patients (F) older than 65 and younger than or equal to 65, (G) patients who were dead and patients who were alive, (H) female patients and male patients, (I) LUAD patients and LUSC patients, (J) patients whose tumor stage were stage I-II and patients whose tumor stage were stage III-IV. The box plot showed that ATscore was not statistically significant in age. (TIF) [file pone.0266070.s004.tif]

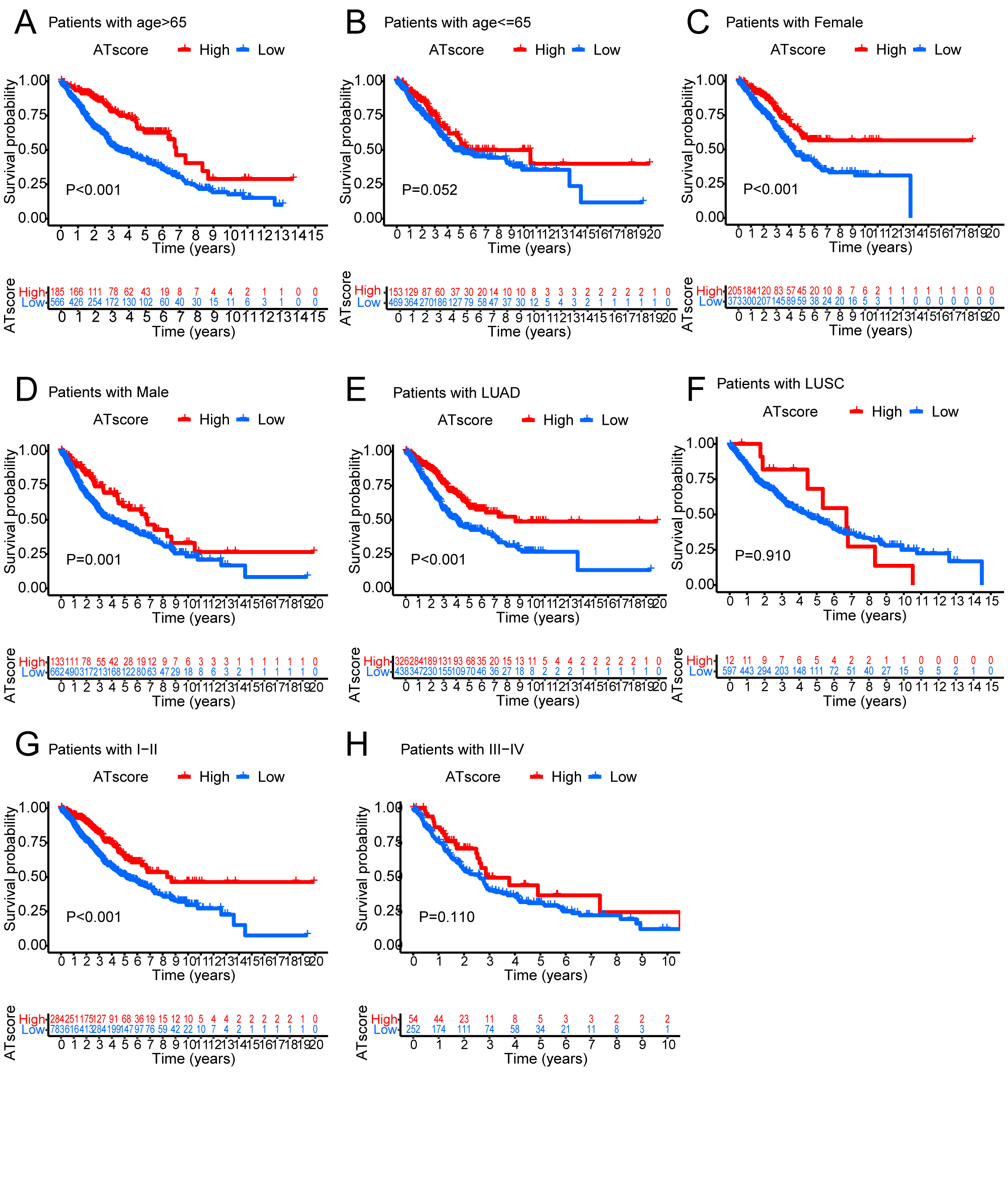

Supplement: S5 Fig — The relationship between OS and autophagy signature in (A) patients over 65 years old, (B) patients younger than or equal to 65, (C) female patients, (D) male patients, (E) LUAD patients, (F) LUSC patients, (G) patients in stage I-II, (H) patients in stage III-IV. The K-M plots showed that the ATscore in patients younger than or equal to 65 and whose tumor pathological were LUSC and whose tumor stage were III-IV was not statistically significant. (TIF) [file pone.0266070.s005.tif]

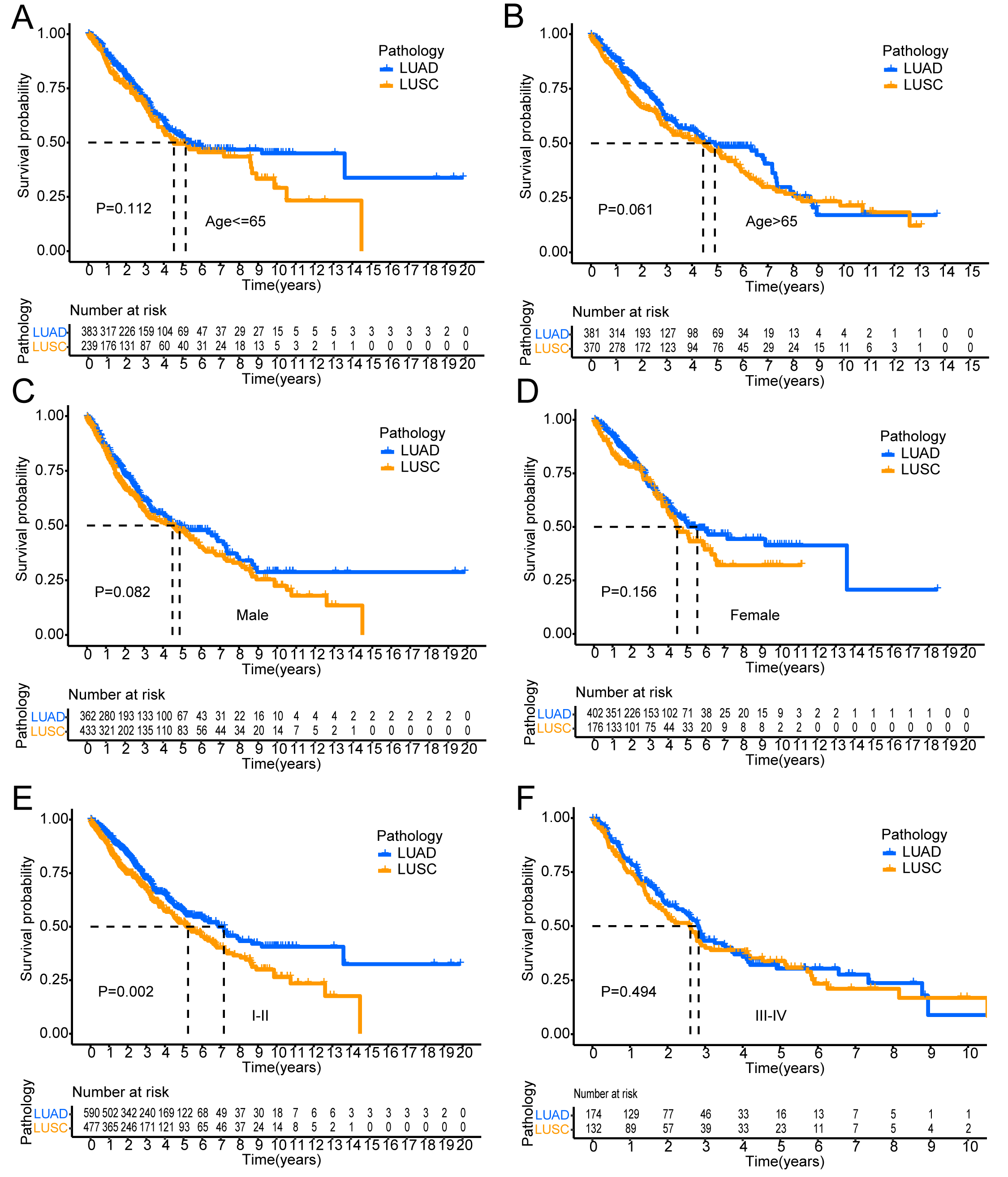

Supplement: S6 Fig — Survival analysis of LUAD and LUSC patients with the same clinical features: (A) age < = 65, (B) age >65, (C) male, (D) female, (E) stage I-II, (F) stage III-IV. K-M curves showed that it was statistically significant when the patient is in stage I-II. Although the remaining P values were greater than 0.05, it could be inferred from the figure that the prognosis of patients with LUAD with the same clinical characteristics was better than that of patients with LUSC. (TIF) [file pone.0266070.s006.tif]

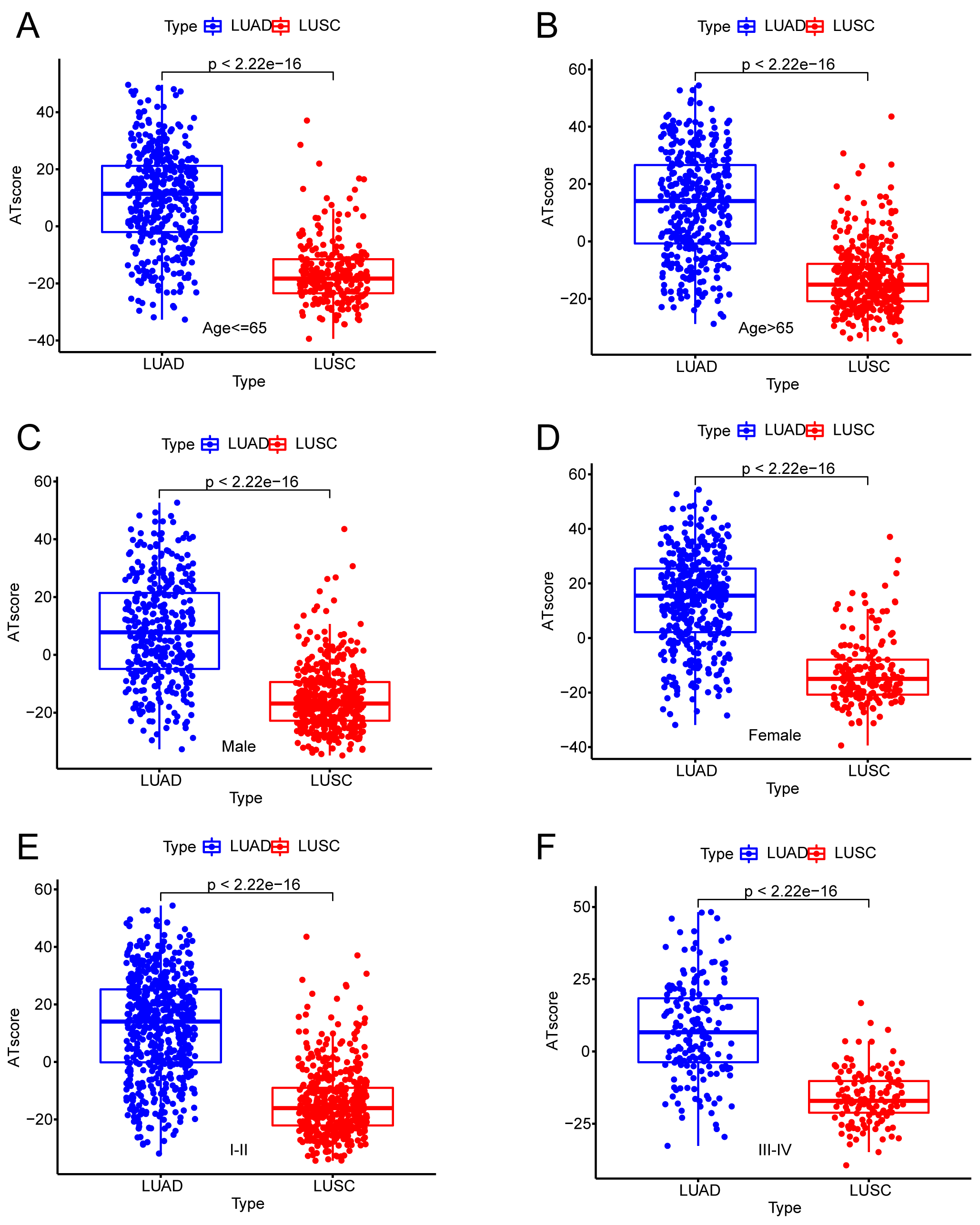

Supplement: S7 Fig — Survival analysis of LUAD and LUSC patients with the same clinical features: (A) age < = 65, (B) age >65, (C) male, (D) female, (E) stage I-II, (F) stage III-IV. K-M curves showed that it was statistically significant when the patient is in stage I-II. Although the remaining P values were greater than 0.05, it could be inferred from the figure that the prognosis of patients with LUAD with the same clinical characteristics was better than that of patients with LUSC. (TIF) [file pone.0266070.s007.tif]

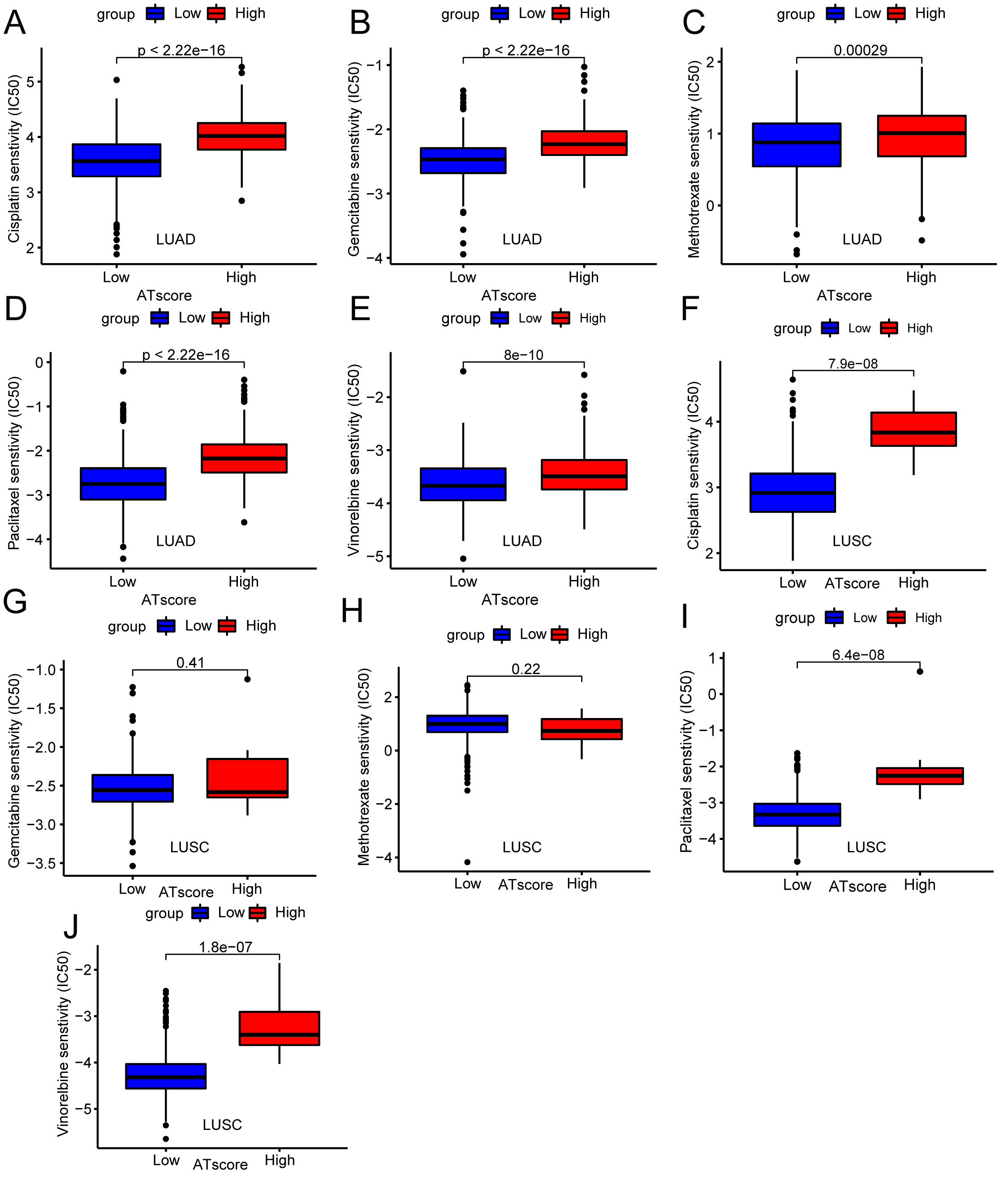

Supplement: S8 Fig — The drug sensitivity of (A, F) cisplatin, (B, G) gemcitabine, (C, H) methotrexate, (D, I) paclitaxel and (E, J) vinorelbine in high and low ATscore groups in LUAD and LUSC. (TIF) [file pone.0266070.s008.tif]
